# Diagnosis of Alzheimer’s disease using plasma biomarkers adjusted to clinical probability

**DOI:** 10.1038/s43587-024-00731-y

**Published:** 2024-11-12

**Authors:** Joseph Therriault, Shorena Janelidze, Andréa Lessa Benedet, Nicholas J. Ashton, Javier Arranz Martínez, Armand Gonzalez-Escalante, Bruna Bellaver, Daniel Alcolea, Agathe Vrillon, Helmet Karim, Michelle M. Mielke, Chang Hyung Hong, Hyun Woong Roh, José Contador, Albert Puig Pijoan, Alicia Algeciras-Schimnich, Prashanthi Vemuri, Jonathan Graff-Radford, Val J. Lowe, Thomas K. Karikari, Erin Jonaitis, Wagner Brum, Cécile Tissot, Stijn Servaes, Nesrine Rahmouni, Arthur C. Macedo, Jenna Stevenson, Jaime Fernandez-Arias, Yi-Ting Wang, Marcel S. Woo, Manuel A. Friese, Wan Lu Jia, Julien Dumurgier, Claire Hourregue, Emmanuel Cognat, Pamela Lukasewicz Ferreira, Paolo Vitali, Sterling Johnson, Tharick A. Pascoal, Serge Gauthier, Alberto Lleó, Claire Paquet, Ronald C. Petersen, David Salmon, Niklas Mattsson-Carlgren, Sebastian Palmqvist, Erik Stomrud, Douglas Galasko, Sang Joon Son, Henrik Zetterberg, Juan Fortea, Marc Suárez-Calvet, Clifford R. Jack, Kaj Blennow, Oskar Hansson, Pedro Rosa-Neto

**Affiliations:** 1 Translational Neuroimaging Laboratory, McGill Research Centre for Studies in Aging, Alzheimer’s Disease Research Unit, Douglas Research Institute, Centre intégré universitaire de santé et de services sociaux de l’Ouest-de-l’Île-de-Montréal, McGill University, Montreal, Quebec Canada; 2 Department of Neurology and Neurosurgery, Faculty of Medicine, McGill University, Montreal, Quebec Canada; 3 Clinical Memory Research Unit, Department of Clinical Sciences Malmö, Lund University, Lund, Sweden; 4 Department of Psychiatry and Neurochemistry, Institute of Neuroscience and Physiology, The Sahlgrenska Academy, University of Gothenburg, Mölndal, Sweden; 5 Wallenberg Centre for Molecular and Translational Medicine, University of Gothenburg, Gothenburg, Sweden; 6 Institute of Psychiatry, Psychology and Neuroscience, Maurice Wohl Clinical Neuroscience Institute, King’s College London, London, UK; 7 NIHR Biomedical Research Centre for Mental Health and Biomedical Research Unit for Dementia at South London and Maudsley NHS Foundation, London, UK; 8 Sant Pau Memory Unit, Department of Neurology, Hospital de la Santa Creu i Sant Pau, Biomedical Research Institute Sant Pau, Universitat Autònoma de Barcelona, Barcelona, Spain; 9 Center of Biomedical Investigation Network for Neurodegenerative Diseases, Madrid, Spain; 10 Barcelonaβeta Brain Research Center, Pasqual Maragall Foundation, Barcelona, Spain; 11 Hospital del Mar Medical Research Institute, Barcelona, Spain; 12 Department of Medicine and Life Sciences, Universitat Pompeu Fabra, Barcelona, Spain; 13 Department of Psychiatry, University of Pittsburgh School of Medicine, Pittsburgh, USA; 14 Institut National de la Santé et de la Recherche Médicale, Université de Paris Cité, Paris, France; 15 Centre de Neurologie Cognitive, Paris, France; 16 Department of Psychiatry, Ajou University School of Medicine, Suwon, Republic of Korea; 17 Division of Epidemiology, Department of Health Sciences Research, Mayo Clinic, Rochester, MN USA; 18 Department of Neurology, Mayo Clinic, Rochester, MN USA; 19 Cognitive Decline and Movement Disorders Unit, Neurology Department, Hospital del Mar, Barcelona, Spain; 20 Department of Medicine, Universitat Autònoma de Barcelona, Barcelona, Spain; 21 ERA-Net on Cardiovascular Diseases Consortium, Barcelona, Spain; 22 Department of Laboratory Medicine and Pathology, Mayo Clinic, Rochester, MN USA; 23 Department of Radiology, Mayo Clinic, Rochester, MN USA; 24 Wisconsin Alzheimer’s Institute, School of Medicine and Public Health, University of Wisconsin–Madison, Madison, WI USA; 25 Wisconsin Alzheimer’s Disease Research Center, School of Medicine and Public Health, University of Wisconsin–Madison, Madison, WI USA; 26 Graduate Program in Biological Sciences: Biochemistry, Universidad Federal do RioGrande do Sul, Porto Alegre, Brazil; 27 Lawrence Berkeley National Laboratory, Berkeley, CA USA; 28 Institute of Neuroimmunology and Multiple Sclerosis, University Medical Center Hamburg–Eppendorf, Hamburg, Germany; 29 San Diego and Shiley-Marcos Alzheimer’s Disease Research Center, University of California, La Jolla, CA USA; 30 Department of Neurology, Skåne University Hospital, Lund University, Lund, Sweden; 31 Wallenberg Center for Molecular Medicine, Lund University, Lund, Sweden; 32 Memory Clinic, Skåne University Hospital, Malmo, Sweden; 33 Clinical Neurochemistry Laboratory, Sahlgrenska University Hospital, Mölndal, Sweden; 34 Department of Neurodegenerative Disease, UCL Institute of Neurology, London, UK; 35 UK Dementia Research Institute UCL, London, UK; 36 Hong Kong Center for Neurodegenerative Diseases, Hong Kong, China; 37 Barcelona Down Medical Center, Fundació Catalana Síndrome de Down, Barcelona, Spain; 38 Centro de Investigación Biomédica en Red de Fragilidad y Envejecimiento Saludable, Madrid, Spain

**Keywords:** Alzheimer's disease, Alzheimer's disease, Ageing

## Abstract

Recently approved anti-amyloid immunotherapies for Alzheimer’s disease (AD) require evidence of amyloid-β pathology from positron emission tomography (PET) or cerebrospinal fluid (CSF) before initiating treatment. Blood-based biomarkers promise to reduce the need for PET or CSF testing; however, their interpretation at the individual level and the circumstances requiring confirmatory testing are poorly understood. Individual-level interpretation of diagnostic test results requires knowledge of disease prevalence in relation to clinical presentation (clinical pretest probability). Here, in a study of 6,896 individuals evaluated from 11 cohort studies from six countries, we determined the positive and negative predictive value of five plasma biomarkers for amyloid-β pathology in cognitively impaired individuals in relation to clinical pretest probability. We observed that p-tau217 could rule in amyloid-β pathology in individuals with probable AD dementia (positive predictive value above 95%). In mild cognitive impairment, p-tau217 interpretation depended on patient age. Negative p-tau217 results could rule out amyloid-β pathology in individuals with non-AD dementia syndromes (negative predictive value between 90% and 99%). Our findings provide a framework for the individual-level interpretation of plasma biomarkers, suggesting that p-tau217 combined with clinical phenotyping can identify patients where amyloid-β pathology can be ruled in or out without the need for PET or CSF confirmatory testing.

## Abstract (web summary)

Therriault et al. provide a framework for the individual-level interpretation of plasma biomarkers by determining their positive and negative predictive values for amyloid positron emission tomography status in relation to patient age and clinical symptoms.

## Main

With the recent Food and Drug Administration approval of disease-modifying therapies for Alzheimer’s disease (AD)^1^, determining eligibility for anti-amyloid-β therapy is an important need for cognitively impaired individuals where AD is a suspected etiology. Anti-amyloid-β immunotherapies currently require evidence of amyloid-β pathology from either positron emission tomography (PET) or cerebrospinal fluid (CSF) to initiate treatment^2^. PET and CSF assessments are limited by cost, accessibility and invasiveness. Minimally invasive, scalable and cost-effective methods to determine the presence of AD pathology are urgently needed^3^.

Several recent studies have reported that plasma biomarkers have excellent diagnostic accuracy for AD, with sensitivity or specificity often exceeding 90% (refs. ^4–10^). However, sensitivity and specificity provide limited information when making decisions about individual patients^11–13^. In contrast, predictive values are critical for interpreting individual-level test results^11,12,14,15^. Sufficiently high positive predictive values (PPVs) or negative predictive values (NPVs) of plasma biomarkers for AD pathology could circumvent the need for the majority of PET or CSF testing, with confirmatory testing used in remaining situations with lower predictive values^3,16^.

Evaluation of the PPVs and NPVs of diagnostic tests in large, unselected populations requires knowledge the prevalence of the disease of interest^11,12,15,17^. As the prevalence of amyloid-β pathology is closely linked to age and clinical syndrome^18–20^, clinical and demographic information can be used to infer the clinical pretest probability of amyloid-β positivity (Aβ+) based on standard clinical assessments^17,21,22^. Here, using the prevalence of amyloid-β pathology from meta-analyses of memory clinic and research settings, we determined the age- and clinical dementia syndrome-associated PPV and NPV of different plasma biomarkers for amyloid-β pathology.

## Results

This study examined a total of 6,896 individuals from Canada, France, South Korea, Spain, Sweden and the United States who were assessed with standardized cognitive assessments, plasma AD biomarkers and established reference standard AD biomarkers (PET, CSF or neuropathological assessments). The mean (s.d.) age of all participants was 69.7 (9.2) years, and 3,698 (53.6%) were female. The mean (s.d.) years of education of the sample was 13.3 (3.6) years. A summary of clinical and demographic characteristics of the entire sample is presented in Table 1, with cohort-specific data presented in Supplementary Tables 3–15. MMSE, Mini-Mental State Examination.

**Table 1:** Demographic and clinical characteristics of the study participants

	Cognitively unimpaired	Cognitively impaired
No.	3,393	3,503
Mean age in years (s.d.)	66.9 (10.6)	72.4 (7.81)
No. of females (%)	1,865 (55.0%)	1,833 (52.3%)
Mean years of education (s.d.)	14.4 (3.18)	12.2 (3.99)
No. of *APOE* ε4 carriers (%)	983 (29%)	1,436 (41%)
Mean MMSE (s.d.)	28.7 (1.35)	24.1 (4.54)
Amyloid-β positive (%)	776 (22.6%)	1,940 (55.4%)

Data are represented as mean and s.d. for continuous variables and number and percentage for categorical variables.

### PPVs and NPVs of plasma biomarkers for Aβ+ in MCI

**Fig. 1:**
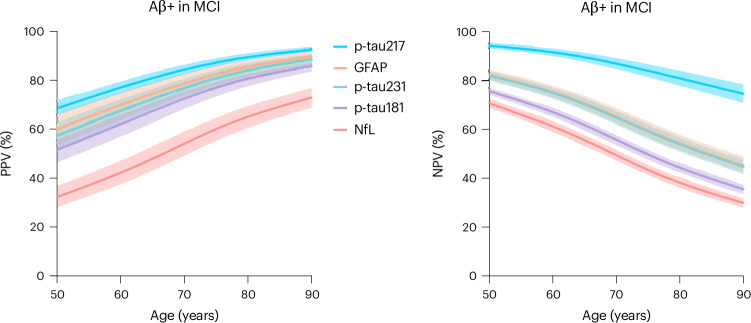
PPVs and NPVs of plasma AD biomarkers in individuals with MCI. Age-associated PPV (left) and NPV (right) of five plasma biomarkers for amyloid PET positivity in MCI. The solid lines represent the point estimate, and error bars represent 95% CIs. Source data

Age-related PPVs and NPVs of five plasma biomarkers for Aβ+ in mild cognitive impairment (MCI) are illustrated in Fig. 1. The ability of plasma biomarkers to rule in or rule out amyloid-β was closely associated with the age-related prevalence of AD pathology in MCI. For individuals with MCI, PPVs of plasma biomarkers increased with age, with p-tau217 reaching 80.9% (95% confidence interval (CI) 78.7–83.1%) at age 65 years and reaching 92.5% (95% CI 91.6–93.5%) for individuals aged 90 years. NPVs for Aβ+ in MCI decreased with age, with NPVs above 90% for individuals younger than 65 years, 80.8% (95% CI 77.8–83.9%) at age 80 years and 74.6% (95% CI 70.9–78.4%) at age 90 years. P-tau181, p-tau231, glial fibrillary acidic protein (GFAP) and neurofilament light chain (NfL) all had lower performance than plasma p-tau217. In *APOE* ε4 carriers with MCI, the PPV of plasma p-tau217 for amyloid-β was higher, reaching 90.8% (95% CI 89.6–91.9%) by age 70 years and 95.6% (95% CI 95.0–96.1%) by age 80 years. Furthermore, in *APOE* ε4 noncarriers with MCI, the NPV of plasma p-tau217 was also higher, being above 95% (95% CI 94.1–96.0%) for individuals aged under 65 years and 89.8% (95% CI 87.9–91.6%) for individuals aged under 80 years. A summary of the PPVs and NPVs of plasma p-tau217 for amyloid PET positivity in all ages and clinical syndromes is presented in Table 2, and a summary of age- and *APOE* ε4-adjusted PPVs and NPVs for individuals with MCI is presented in Supplementary Tables 16–18.

**Table 2:** PPVs and NPVs of plasma p-tau217 for amyloid-β pathology in different clinical syndromes

	MCI		Probable AD dementia		Frontotemporal dementia		Vascular dementia		Corticobasal syndrome	
Age in years	PPV % (95% CI)	NPV % (95% CI)	PPV % (95% CI)	NPV % (95% CI)	PPV % (95% CI)	NPV % (95% CI)	PPV % (95% CI)	NPV % (95% CI)	PPV % (95% CI)	NPV % (95% CI)
50–54	68.4% (65.5–71.4%)	94.4% (93.3–95.4%)	97.5% (96.5–98.1%)	44.6% (40.1–50%)	27.6% (25–30.5%)	99% (98.8–99.2%)	45.2% (41.9–48.7%)	97.8% (97.3–98.2%)	Prevalence data unavailable	Prevalence data unavailable
55–59	73.1% (70.5–75.9%)	93% (91.7–94.3%)	97.4% (96.3–98%)	45.7% (41.2–51.1%)	30.5% (27.8–33.7%)	98.8% (98.6–99%)	49.1% (45.8–52.7%)	97.4% (96.9–97.9%)	82.9% (81–84.9%)	88.2% (86.1–90.3%)
60–64	77.1% (74.7–79.6%)	91.5% (89.9–93%)	97.3% (96.1–97.9%)	47% (42.4–52.4%)	33.3% (30.5–36.6%)	98.6% (98.4–98.9%)	52.6% (49.3–56.2%)	97% (96.4–97.6%)	81.2% (79.1–83.3%)	89.4% (87.5–91.3%)
65–69	80.9% (78.7–83%)	89.6% (87.7–91.4%)	97% (95.7–97.7%)	49.5% (44.8–54.8%)	40.8% (37.7–44.3%)	98.1% (97.8–98.5%)	57.3% (54.1–60.8%)	96.4% (95.7–97.1%)	76.4% (73.9–78.9%)	91.8% (90.3–93.3%)
70–74	84% (82.2–85.9%)	87.3% (85.1–89.5%)	96.7% (95.4–97.5%)	51.7% (47.1–57.1%)	47.2% (43.9–50.7%)	97.6% (97.1–98.1%)	64% (60.9–67.2%)	95.3% (94.4–96.2%)	74% (71.4–76.7%)	92.7% (91.4–94.1%)
75–79	87.5% (86–89%)	83.9% (81.2–86.6%)	96.5% (95–97.3%)	53.9% (49.3–59.2%)	52.6% (49.3–56.2%)	97% (96.4–97.6%)	69.4% (66.6–72.4%)	94.1% (93–95.2%)	69.4% (66.6–72.4%)	94.1% (93–95.2%)
80–84	89.6% (88.3–90.9%)	80.8% (77.8–83.9%)	96.2% (94.6–97.1%)	55.9% (51.3–61.1%)	57.3% (54.1–60.8%)	96.4% (95.7–97.1%)	74% (71.4–76.7%)	92.7% (91.4–94.1%)	64% (60.9–67.2%)	95.3% (94.4–96.2%)
85–89	91.1% (90–92.2%)	77.9% (74.6–81.4%)	95.7% (93.9–96.7%)	58.6% (54.1–63.7%)	61.5% (58.3–64.8%)	95.8% (95–96.6%)	77.8% (75.5–80.2%)	91.2% (89.6–92.8%)	60.2% (57–63.6%)	96% (95.2–96.8%)
90–95	92.5% (91.6–93.5%)	74.6% (70.9–78.4%)	95.3% (93.3–96.4%)	61.2% (56.7–66.1%)	65.2% (62.1–68.3%)	95.1% (94.2–96%)	81.2% (79.1–83.3%)	89.4% (87.5–91.3%)	55.8% (52.6–59.3%)	96.6% (96–97.3%)

### PPVs and NPVs of plasma biomarkers for Aβ+ in probable AD dementia

**Fig. 2:**
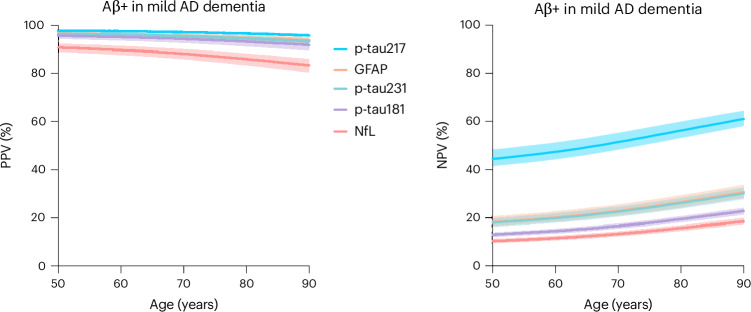
PPVs and NPVs of plasma biomarkers of AD in individuals with probable AD dementia. Age-associated PPV (left) and NPV (right) of five plasma biomarkers for amyloid PET positivity in probable AD dementia. The solid lines represent the point estimate, and error bars represent 95% CIs. Source data

Age-associated PPVs and NPVs of five AD plasma biomarkers in probable AD dementia are reported in Fig. 2. In individuals with probable AD dementia, plasma biomarkers, particularly p-tau217, had very high PPVs (above 95%) for Aβ+ at all ages. Owing to the high prevalence of Aβ+ in individuals with probable AD dementia, NPVs of plasma biomarkers was comparatively lower. Again, p-tau217 had the highest NPV at all age ranges for individuals with probable AD dementia, reaching 60% by age 90 years. Other plasma biomarkers had lower NPVs at all ages. A summary of age- and *APOE* ε4-adjusted PPVs and NPVs for individuals with probable AD dementia is presented in Supplementary Tables 19–21.

### PPVs and NPVs of plasma biomarkers for Aβ+ in non-AD clinical syndromes

In non-AD dementia syndromes, plasma biomarkers, in particular p-tau217, could rule out the presence of AD pathology with NPVs above 90% in nearly all circumstances. Two exceptions to this were ruling out amyloid-β pathology in individuals with vascular dementia above age 90 years (NPV 89.4%, 95% CI 87.5–91.3%) and ruling out amyloid-β pathology in individuals with corticobasal syndrome younger than age 65 years (NPV 88.2%, 95% CI 86.1–90.3%). A summary of the PPVs and NPVs of plasma p-tau217, the best-performing biomarker, for amyloid PET positivity in all ages and clinical syndromes is presented in Table 2. A summary of PPVs and NPVs of plasma biomarkers for amyloid-β pathology additionally adjusted for the *APOE* ε4 genotype in non-AD dementia syndromes is presented in Supplementary Tables 22–33.

## Discussion

This study evaluated the PPVs and NPVs of plasma biomarkers for amyloid-β pathology in relation to patient age and clinical syndrome. We report that, in older adults with MCI (ages 80+ years) or in individuals with clinically diagnosed probable AD dementia, plasma p-tau217 can rule in amyloid-β pathology with PPVs above 90%. Furthermore, in non-AD dementia syndromes such as frontotemporal dementia, vascular dementia and corticobasal syndrome, plasma p-tau217 could rule out AD pathology with NPVs above 90%. Owing to the high prevalence of amyloid-β pathology in individuals with clinically diagnosed AD dementia, negative plasma biomarkers will warrant confirmatory testing to rule out AD pathology in individuals with these symptoms. Similarly, in older adults with MCI where the prevalence of AD pathology is high, confirmatory testing is needed to rule out AD pathology. Taken together, our study provides a framework for the individual-level interpretation of plasma biomarkers for AD according to patient age and clinical syndrome^23^.

The PPVs and NPVs reported in the present study are to be understood within the context of the prevalence of amyloid-β pathology within MCI, probable AD dementia and other non-AD dementia syndromes. MCI is a highly heterogeneous clinical syndrome that can be caused by several different neurodegenerative and nonneurodegenerative conditions^24^. Estimates from memory clinic and community-based studies suggests the prevalence of amyloid-β pathology in individuals with MCI is relatively low for individuals in their 60s but reaches 75–80% by age 90 years^19,20^. Correspondingly, the PPV of plasma p-tau217 for the detection of amyloid-β pathology in MCI rose with age, exceeding 95% by age 90 years. Owing to the high pretest probability that amyloid-β is present in older adults with MCI, the NPV of even highly accurate plasma biomarkers fell below 80% with more advanced age.

The clinical syndrome of probable AD dementia is more closely associated with amyloid-β pathology than MCI at all ages^19,20,25^ Therefore, in clinically diagnosed probable AD dementia, the PPV of plasma biomarkers, particularly p-tau217, is very high and probably sufficient to rule in amyloid-β pathology. The corollary is that the NPV of plasma biomarkers for AD was lower owing to the high prevalence of AD pathology in this clinical syndrome. Studies in other areas of medicine have also found lower NPVs of even highly sensitive and specific tests in situations where the pretest probability of a disease is high^23,26,27^. The risk of a false negative in probable AD dementia may be high enough to warrant confirmatory CSF or PET testing for individuals with clinically diagnosed probable AD dementia with a negative plasma biomarker test result, even for highly accurate biomarkers such as plasma p-tau217.

Owing to the substantially higher prevalence of Aβ+ in *APOE* ε4 carriers^18,19,28^, plasma biomarkers had higher PPVs for brain amyloid-β, particularly in individuals with MCI. Conversely, the NPV of plasma biomarkers, particularly p-tau217, was substantially higher in *APOE* ε4 noncarriers. Therefore, genotyping for *APOE* (also available with a blood sample) will lead to higher predictive values for Aβ+.

Across all cohorts and assays investigated, a consistent finding in this study is that plasma p-tau217 had the highest PPVs and NPVs for amyloid-β pathology. These results are consistent with a number of recent studies demonstrating excellent performance of multiple p-tau217 assays in the differential diagnosis of cognitive impairment^8–10,29–32^, its close association with amyloid-β and tau pathologies^33,34^ and longitudinal increases over time in Aβ+ individuals^35^. Plasma GFAP had slightly lower performance than p-tau217, with notably lower specificity. Despite the role of GFAP in AD pathogenesis^36^ and in predicting future dementia incidence^37^, the lower specificity of GFAP may limit its role as a diagnostic biomarker for AD^38^. For example, GFAP elevations have been reported in frontotemporal dementia^39^, traumatic brain injury^40^, multiple sclerosis^41^ and inflammatory central nervous system diseases^42^. Despite these limitations, GFAP nonetheless performed better overall than other plasma biomarkers such as p-tau181. However, it is important to emphasize that head-to-head studies indicate that different assays for p-tau181 vary substantially in their diagnostic performance^9,10^ and may not all perform inferiorly to GFAP in all contexts^34,43^. As expected, plasma NfL had relatively lower PPV and NPV for AD, as NfL is a nonspecific biomarker of neurodegeneration, elevated in multiple different neurodegenerative diseases^44^. Taken together, these results highlight the utility of plasma p-tau217 for the differential diagnosis of cognitive impairment and for determining eligibility for anti-amyloid-β disease-modifying therapies.

Currently, anti-amyloid monoclonal antibodies require the confirmation of amyloid-β pathology from PET or CSF before initiating therapy^45,46^. On the basis of the present results, plasma biomarkers, particularly plasma p-tau217, may be suitable to rule in amyloid-β pathology in individuals with probable AD dementia or in older adults with MCI, which stands to circumvent a large number of PET scans or lumbar punctures. In contrast, in non-AD clinical syndromes such as frontotemporal dementia, vascular dementia and corticobasal syndrome, which are less frequently associated with AD pathology^18^, plasma biomarkers can rule out AD pathology at almost all ages. As the prevalence of AD pathology is associated with age in non-AD syndromes^18^, the PPV and NPV of plasma biomarkers also varies slightly with age. For example, because of the relatively higher prevalence of AD pathology in younger individuals with corticobasal syndrome^18^, caution is warranted in using plasma biomarkers to rule out AD in these individuals. Overall, however, plasma biomarkers are more limited in ruling in AD pathology in non-AD clinical syndromes and follow-up testing with either PET or CSF may be warranted; in these instances, the topographical information provided by tau-PET^47,48^ may be useful. Plasma biomarkers may therefore have an important role in reducing the patient burden associated with the initiation of anti-amyloid-β therapies for AD, which at present require biomarker confirmation with PET or CSF, as well as serial magnetic resonance imaging to monitor for adverse events^45,46^. However, it is also important to consider that multiple neuropathological processes are often present in older individuals with cognitive symptoms, and plasma biomarkers alone cannot determine whether AD is the driving force behind a specific clinical syndrome. This is especially true of biomarkers that plateau in later disease stages^49^. In the future, plasma biomarker panels that measure p-tau217 in addition to biomarkers that become abnormal at later stages such as p-tau205 (refs. ^50,51^) or MTBR-tau243 (ref. ^52^) may prove beneficial in this regard^53^. Furthermore, more work is needed to determine what is an acceptable PPV for Aβ+ for the initiation of anti-amyloid therapy, as it is possible that PPVs below 85–90% may not be sufficient and more invasive/expensive testing may be warranted.

The results of our study used amyloid-β pathology prevalence estimates derived from the Amyloid Biomarker Study Group, an international multicenter study of more than 19,000 individuals^19,28^. These prevalence estimates informed the age-associated pretest probability of amyloid-β pathology in MCI, probable AD dementia and non-AD dementia clinical syndromes, which permit PPVs and NPVs to be estimated^12,17^. These prevalence estimates are largely based on subjects recruited from clinical and research settings that feature some enrollment biases and are not representative in terms of race or ethnicity of the populations at risk for dementia globally. Furthermore, research-level phenotyping may result in stronger clinico-pathological correlations in individuals with MCI, AD dementia and non-AD syndromes than can be reasonably achieved in nonspecialist centers. However, very similar results were observed when using prevalence estimates from the Mayo Clinic Study of Aging, a population-based cohort study^20^.

Performance of specific plasma AD biomarkers was overall highly comparable across different centers, settings and populations. For example, the sensitivity and specificity of p-tau181 in the Health and Aging Brain Study–Health Disparities (HABS-HD) cohort, a multiethnic and multiracial community-based research study, which features a high proportion of Mexican–American and African–American individuals, was nearly identical to p-tau181 performance in highly specialized memory clinic settings. While p-tau217 was not available in some cohorts, previous studies have provided evidence that this biomarker also has excellent performance in different racial and ethnic groups^54,55^. Our study contributes to this finding by providing evidence of excellent diagnostic performance of plasma p-tau217 for AD in a large multicenter memory clinic cohort from South Korea.

Our study has important limitations. First, the binary classification of individuals into categories based on the presence/absence of disease is a limitation; it is anticipated that plasma biomarker accuracy is higher in later-stage disease when burden of pathology is greater. Second, while our study used a standardized method of determining plasma biomarker abnormality across centers, future work may be able to further optimize this method, in turn providing higher PPVs and NPVs. For example, recent evidence suggests that a three-range method leads to higher accuracy to identify amyloid PET positivity in individuals with MCI^56^. Third, the use of plasma biomarker ratios may further improve accuracy by circumventing associations between chronic kidney disease and elevated plasma biomarker concentrations^57^. Fourth, refinements to the clinical pretest probability estimates (for example, through polygenetic risk scores^58^ or through basic algorithms incorporating age, *APOE* genotype and cognitive testing^59^) will probably further improve plasma biomarker diagnostic performance and interpretation. Fifth, our study is a cross-sectional diagnostic study and is not designed to predict who will develop AD dementia in the future. Blood biomarkers of amyloid-β misfolding have shown promise in this regard^60,61^. Sixth, the amyloid PET positivity prevalence estimates employed in our study are derived from meta-analyses of predominantly memory clinic and research settings^18,19^. Correspondingly, the PPV and NPV estimates from our study should not be extrapolated to other clinical settings where the prevalence of AD is substantially different^11,12,14,15^.

In conclusion, our study provides information about the interpretation of plasma biomarkers for AD at the individual level, adjusted to clinical pretest probability. Our study provides evidence that, in individuals with probable AD dementia and in older individuals with MCI, plasma biomarkers can be used to rule in amyloid-β pathology, required for the initiation of disease-modifying therapies. In individuals with non-AD dementia syndromes, a negative plasma p-tau217 result can rule out AD pathology, with follow-up testing required for non-AD dementia syndrome cases with a positive AD plasma biomarker.

## Methods

### Study patients

This study evaluated individuals assessed with standardized cognitive assessments, plasma biomarkers of AD and reference standard AD biomarker assessments (either PET, CSF or neuropathological assessments). Patients were enrolled from prospective cohort studies in Canada, France, South Korea, Spain, Sweden and the United States. AD biomarker abnormality was not required for enrollment in any of the participating sites. All study participants provided written informed consent, and local institutional review boards approved the studies. A detailed description of inclusion and exclusion criteria for all prospective cohort studies is provided in the Supplementary Appendix.

### Plasma biomarker assessments

The plasma biomarkers evaluated in this study were p-tau181, p-tau217, p-tau231, GFAP and NfL. Assays for p-tau181 included the in-house assay from the University of Gothenburg and from Quanterix. Assays of p-tau217 included assays from Lilly, Janssen and ALZPath. Plasma p-tau231 was assessed using the in-house assay developed at the University of Gothenburg. GFAP and NfL concentrations were measured using the Quanterix assay. The details of all assays can be found in the Supplementary Information.

### Reference standard biomarker assessments

The reference standards used in this study to determine the presence of AD pathology were PET, CSF and neuropathological assessments. Abnormality criteria for all reference standard biomarkers have been published previously and are described in the Supplementary Information for all cohorts.

### Statistics and reproducibility

Abnormality for plasma biomarkers was determined in a standardized manner across all cohorts using *z*-scores created based on the means and s.d. of cognitively unimpaired individuals without elevated amyloid-β pathology, as previously done in several studies^8,62,63^. These *z*-scores were applied to the cognitively impaired individuals with reference standard biomarkers assessed by dementia specialists. In the TRIAD cohort and McGill memory clinic cohorts, a *z*-score of 1.5 had high discriminative accuracy for biological AD versus other neurodegenerative diseases. Therefore, plasma biomarker abnormality was defined by a *z*-score of 1.5 and above, and this was applied consistently to all cohorts. Prevalence-adjusted (that is, pretest probability-adjusted) PPVs and NPVs were calculated using the Bayesian formula provided by Altman and Bland^15,64,65^ using age-associated prevalence of Aβ+ in MCI, probable AD dementia and non-AD dementia syndromes (frontotemporal dementia, vascular dementia and corticobasal syndrome) from published meta-analyses^18,19^ using the following formulas:

$${\mathrm{PPV}}=\frac{\mathrm{sensitivity}\times {\mathrm{prevalence}}}{({\mathrm{sensitivity}}\times {\mathrm{prevalence}})+((1-{\mathrm{specificity}})\times (1-{\mathrm{prevalence}}))},$$

$${\mathrm{NPV}}=\frac{\mathrm{specificity}\times (1-{\mathrm{prevalence}})}{((1-{\mathrm{sensitivity}})\times {\mathrm{prevalence}})+({\mathrm{specificity}}\times (1-{\mathrm{prevalence}}))}.$$

We furthermore conducted three sets of sensitivity analyses. First, owing to the strong association of *APOE* ε4 genotype with amyloid-β pathology^18,19^, we estimated age- and clinical syndrome-associated plasma biomarker PPVs and NPVs adjusted for *APOE* ε4 carriership. In the second, we estimated PPVs and NPVs using the upper and lower estimates of the reported prevalence of amyloid-β pathology^18,19^. In the third, we used prevalence estimates of amyloid PET positivity from the Mayo Clinic Study of Aging, a population-based cohort study^20^. No statistical methods were used to predetermine sample sizes. No data were excluded from any of the analyses. Data were visualized using GraphPad Prism (version 10). This study complied with Standards for Reporting Diagnostic Accuracy Studies guidelines.

### Reporting summary

Further information on research design is available in the Nature Portfolio Reporting Summary linked to this article.

## Supplementary information

Supplementary Information Supplementary Methods and Tables 1–45.

Reporting Summary

## Source data

Source Data Fig. 1 Point estimates and confidence intervals for PPVs and NPVs for individuals with MCI.

Source Data Fig. 2 Point estimates and confidence intervals for PPVs and NPVs for individuals with probable AD dementia.

## Data Availability

Data from the ADNI cohort can be accessed from https://ida.loni.usc.edu. Data from the HABS-HB study can be accessed from https://apps.unthsc.edu/itr/researchers. Raw and analyzed de-identified data from the Mayo Clinic Study of Aging can be requested at https://ras-rdrs.mayo.edu/Request/IndexRequest. The request will be reviewed by the Mayo Clinic Study of Aging investigators and Mayo Clinic to verify whether the request is subject to any intellectual property or confidentiality obligations. A data sharing agreement must be obtained before release. Anonymized data from the BICWALZS, BioCogBank, BIODEGMAR, BioFINDER, SPIN, TRIAD and UCSD-ADRC cohort studies will be shared by request from a qualified academic investigator for the sole purpose of replicating procedures and results presented in this Letter and as long as the data transfer is in agreement with all local legislation on general data protection regulation and will be regulated by a material transfer agreement. Source data are provided with this paper.

## Code availability

No custom code or mathematical algorithm that was central to the conclusions was used for this study.
