# Supplementary material for: Diagnosis of Alzheimer’s disease using plasma biomarkers adjusted to clinical probability
Source: Nat Aging. 2024 Nov 12;4(11):1529–37. doi: 10.1038/s43587-024-00731-y (PMC11564087; doi:10.1038/s43587-024-00731-y)
Supplement: Supplementary file 1 — Supplementary Methods and Tables 1–45. [file 43587_2024_731_MOESM1_ESM.pdf]

nature aging

Supplementary information https://doi.org/10.1038/s43587-024-00731-y

Diagnosis of Alzheimer's disease using plasma biomarkers adjusted to clinical probability

In the format provided by the authors and unedited

Supplementary Appendix

Supplement to: Diagnosis of Alzheimer's disease using plasma biomarkers adjusted to clinical probability

This appendix has been provided by the authors to give readers additional information about the work.

Contents:

Supplemental methods: Individual cohort study information.

Supplemental table 1: Representativeness of the study.

Supplemental table 2: Comparative characteristics of the study cohorts.

Supplemental tables 3-15: clinical and demographic characteristics of individual study cohorts.

Supplemental tables 22-26 (pages 36-40): PPV and NPV of different plasma biomarkers for amyloid-β positivity according to age and clinical syndrome in APOEε4 noncarriers.

Supplemental tables 27-31: PPV and NPV of different plasma biomarkers for amyloid-β positivity according to age and clinical syndrome in APOEε4 carriers.

Supplemental table 32: PPV and NPV of different plasma p-tau217 for amyloid-β positivity according to age and clinical syndrome in APOEε4 carriers.

Supplemental table 33: PPV and NPV of different plasma p-tau217 for amyloid-β positivity according to age and clinical syndrome in APOEε4 carriers.

Supplemental tables 34-38: PPV and NPV of different plasma biomarkers for amyloid-β positivity using lower estimates of amyloid-β positivity prevalence.

Supplemental tables 39-43: PPV and NPV of different plasma biomarkers for amyloid-β positivity using higher estimates of amyloid-β positivity prevalence.

Supplemental Table 44: PPV and NPV of plasma biomarkers for amyloid-β positivity in MCI using amyloid-β positivity prevalence estimates from a community-based setting.

Supplemental Table 45: Proportion of individuals with MCI who are amyloid-β positive in each cohort.

Participant characteristics and biomarker assessments

Alzheimer's Disease Neuroimaging Initiative

Participants

We examined a total 1092 individuals from the Alzheimer's Disease Neuroimaging Initiative cohort (ADNI), a North-American multisite cohort launched in 2003 by the National Institute on Aging, the National Institute of Biomedical Imaging and Bioengineering, the Food and Drug Administration, private pharmaceutical companies, and nonprofit organizations. Subjects were between ages 55 and 90 years at baseline. Participants were evaluated by dementia specialists with standard clinical assessments. Participants with a Clinical Dementia Rating (CDR) of 0 were categorized as being Cognitively Unimpaired (CU). Participants with mild cognitive impairment (MCI) and had a CDR of 0.5 and met standard criteria [1], and individuals with AD dementia had a CDR score of 1 or 2 and met standard clinical diagnostic criteria for AD dementia [2]. In all cases, clinical diagnoses were made before biomarker assessments. Full information regarding the ADNI inclusion and exclusion criteria, as well as a complete study protocol, is available at http://adni.loni.usc.edu/ (accessed August 2023). Briefly, exclusion criteria included any serious neurological disease other than possible AD, any history of significant brain lesions or head trauma, or psychoactive medication use (including antidepressants, neuroleptics, chronic anxiolytics, or sedative hypnotics).

Biomarker assessments

901 participants had CSF assessments of Aβ42 and p-tau181 as the reference standard and 191 additional individuals had amyloid-PET with [^{18}F]Florbetapir as the reference standard. ADNI PET acquisition and processing and CSF p-tau$_{181}$ quantification have been described previously [3]. Briefly, CSF samples were obtained in the morning after an overnight fast. Aliquots (0.5ml) were prepared from these samples after thawing (1 hour) at room temperature and gentle mixing. The aliquots were stored in bar code labeled polypropylene vials at -80°C. Plasma biomarker analysis of p-tau181 and NfL has also been described previously [4]. The ADNI study was approved by the Institutional Review boards of all participating institutions. All participants provided informed written consent.

Biobank Innovations for chronic Cerebrovascular disease With ALZheimer's disease Study

Participant information

This study used data from the ongoing Biobank Innovations for chronic Cerebrovascular disease With ALZheimer's disease Study (BICWALZS) and the Centre for Convergence Research of Neurological Disorders. The BICWALZS was initiated in October 2016 as part of the Korea Biobank Project, a national program aimed at promoting biomedical and healthcare research and development infrastructure through innovative biobanking. The study involved memory clinics from five university hospitals and a community geriatric mental health center. Participants were recruited on a voluntary basis from individuals who visited these neurology or psychiatry memory outpatient clinics. The primary objective was to facilitate, regulate, and ensure the optimal utilization of human biological specimens for research, utilizing real-world data in the areas of SCD, MCI, AD, and subcortical vascular dementia (SVaD). For this study, specific clinical diagnostic criteria were utilized as follows: Subjective Cognitive Decline (SCD) criteria involved reports of cognitive decline by the individual and/or their informant, but without any objective impairment in cognitive tasks (scoring no less than -1.5 standard deviations in each neurocognitive test domain and having a Clinical Dementia Rating of 0). Patients with Mild Cognitive Impairment (MCI) were assessed using a Clinical Dementia Rating (CDR) score of 0.5 [5] along with the expanded Mayo Clinic criteria. Patients diagnosed with Alzheimer's Disease (AD) dementia were evaluated based on the National Institute on Aging-Alzheimer's Association core clinical probable AD dementia criteria [6]. Subcortical Vascular Dementia (SVaD) was evaluated based on above-moderate white matter hyperintensity (WMH) and vascular dementia criteria following the guidelines of the Diagnostic Statistical Manual of Mental Disorders, fifth edition. Patients with a history of neurological or medical conditions such as territorial cerebral infarction, intracranial hemorrhage, Parkinson's disease, heart failure, renal failure, or other conditions that could potentially interfere with the study were excluded from participation.

The BICWALZS has been officially recorded in the Korean National Clinical Trial Registry under the identifier KCT0003391. This research received approval from the Institutional Review Board of Ajou University Hospital with the reference number AJIRB-BMR-SUR-16-362. All participants and caregivers provided written informed consent before taking part in the study.

Recruitment of participants for the BICWALZS took place at memory clinics situated in seven university-affiliated hospitals and community geriatric centers across South Korea.

Biomarker assessments

Subjects underwent amyloid-PET scans using [^{18}F]flutemetamol and a Discovery Ste/690 PET/CT scanner (GE, Milwaukee, WI, USA). After a 90-minute interval, a PET scan lasting 20 minutes (comprising 4 sets of 5-minute dynamic frames) was performed. These [^{18}F]flutemetamol PET scans were aligned with individual MRI scans, which were standardized to a T1-weighted MRI template. The MRI-aligned [^{18}F]flutemetamol PET images were then conformed to the MRI template through transformation parameters. For quantifying the retention of [^{18}F]flutemetamol, the standard uptake value ratio (SUVR) was computed using the pons as a reference area. The accumulation of [^{18}F]flutemetamol in the overall cortex was computed by determining the volume-weighted average SUVR across ten specified cortical regions of interest. These regions encompassed the frontal, posterior cingulate, lateral temporal, parietal, and occipital lobes, and the annotated anatomical labeling (AAL) atlas was utilized for this purpose. In accordance with findings from a previous study on elderly individuals in Korea and our own observed data patterns, participants were categorized as having amyloid presence if their global cortical SUVR exceeded 0.634 as previously described [7]. Blood specimens were gathered in the morning following an overnight fast using venipuncture. These specimens were placed into tubes designed for serum separation and tubes containing dipotassium ethylenediaminetetraacetic acid. Baseline standard blood tests included plasma p-tau217 with the ALZpath assay [8], NfL, and apolipoprotein E (APOE) genotype. The blood samples were stabilized and then subjected to centrifuged for 10 minutes at room temperature, resulting in the separation of plasma and serum supernatants. The resultant samples were collected and promptly stored in a deep freezer at -80°C.

BioCogBank memory clinic cohort

Participants

The BioCogBank Paris Lariboisière cohort was provided by the Center of Cognitive Neurology, University Hospital Lariboisière Fernand Widal, a centre specializing in managing patients with cognitive disorders and neurodegenerative diseases. The cohort comprised individuals who visited the center with complaints related to neurocognitive issues and underwent

neuropsychological assessment, MRI, and CSF analysis. The final clinical diagnoses of Alzheimer's disease (AD), Mild Cognitive Impairment (MCI), and other neurodegenerative diseases were reached through discussions among neurologists, geriatricians, neuroradiologists, biochemists and neuropsychologists, adhering to the diagnostic criteria for MCI [1] and AD [6] or other neurodegenerative diseases. Control participants sought medical attention for cognitive concerns or were part of observational research studies. They were categorized as cognitively unimpaired (CU) when the neurologist ruled out any neurocognitive disorder and the neuropsychological assessment showed preserved global cognition, with scores in the normative range considering age, sex, and level of education.

Biomarker assessments

Within the BioCogBank Paris Lariboisière group, venous and lumbar punctures were executed subsequent to an overnight fasting period. Cerebrospinal fluid (CSF) was obtained for analytical purposes, then subjected to centrifugation at a temperature of 4°C. It was promptly divided into smaller portions and stored at a temperature of -80°C until the time of analysis. The plasma, on the other hand, underwent centrifugation at 2000g for a duration of 20 minutes, all at a temperature of 4°C. The resultant plasma supernatant was gathered and maintained at a temperature of -80°C for subsequent usage. The Lumipulse G1200 assay system from Fujirebio was employed to analyze the CSF biomarkers. In the BioCogBank cohort, individuals were deemed to have a CSF profile compatible with AD if the Aβ42/40 ratio < 0.068 and p-tau181 values above 50 pg/ml [9]. In the BioCogBank cohort, p-tau181, and p-tau231 were analyzed using assays from the University of Gothenburg, and GFAP and NfL were analyzed using assays from Quanterix [41].

BIODEGMAR memory clinic cohort

Participant information

The BIODEGMAR cohort is a prospective observational study of patients with neurodegenerative diseases evaluated in the Cognitive Decline and Movement Disorders Unit of Hospital del Mar in Barcelona, Spain. Participants in the cohort donated blood, had neurological and neuropsychological evaluations, and had brain magnetic resonance imaging. Clinical evaluations were conducted by a neurologist, which included gathering medical history,

performing a physical examination, and making a clinical diagnosis. Neuropsychological evaluations were carried out by a neuropsychologist; the evaluation consists of specific cognitive tests and functional scales, including the Mini Mental State Examination, Memory Impairment Screen, Automatic reverse series (a subtest of the Barcelona cognitive battery), Semantic fluency task (another subtest of the Barcelona cognitive battery), Free and Cued Selective Reminding Test, Boston Naming Test, Trail Making Test, Blessed Dementia Rating Scale, and Alzheimer's Disease Functional Assessment and Change Scale.

Biomarker assessments

CSF collection has been described previously [10]. Individuals were deemed to have a CSF profile compatible with AD if the Aβ42/p-tau$_{181}$ < 10.25, and a non-AD CSF profile with a Aβ42/p-tau$_{181}$ ratio >= 10.25 as reported previously [10]. In the BIODEGMAR cohort, p-tau181 and p-tau231 were analyzed using assays from the University of Gothenburg, p-tau217 was analyzed using the assay from Lilly, and GFAP and NfL were analyzed using assays from Quanterix as described previously [10].

BioFINDER-2 Study

Participant information

In the current study, we included CU individuals and those with MCI, AD dementia and other neurodegenerative disorders from the prospective Swedish BioFINDER-2 study (NCT03174938) who had plasma biomarker, CSF Aβ42/Aβ40 data available (dates of enrollment: April 2017-April 2022). The BioFINDER-2 study enlists participants at Skåne University Hospital and the Hospital of Ängelholm in Sweden comprising individuals without cognitive impairments (either no cognitive concerns or subjective cognitive decline [SCD]) and those with MCI, dementia due to AD and other neurodegenerative disorders. Participants referred to memory clinics due to cognitive symptoms are classified as having MCI if their performance in any cognitive domain was more than 1.5 standard deviations below the mean based on age and education-adjusted test norms [11]. Those who do not meet the criteria for MCI are categorized as having SCD. Diagnosis of dementias and neurodegenerative disorders are established using the Diagnostic and Statistical Manual of Mental Disorders Fifth Edition [DSM-5] criteria and additionally amyloid positivity by PET or CSF is required for diagnosis of AD dementia. The inclusions and exclusion criteria for the Swedish BioFINDER-2 study have been published in detail elsewhere [11,12].

Biomarker assessments

CSF and blood samples were drawn in the morning while participants were not necessarily non-fasting and handled as previously described [13,14]. CSF was processed according to current international recommendations [15]. Plasma samples from all 1373 study participants were analyzed for p-tau217 using Lilly MSD assay at Lund University and for NfL and GFAP using Quanterix assay at the University of Gothenburg. Plasma p-tau181 and p-tau231 were quantified using in-house assays at the University of Gothenburg in 717 and 1342 participants, respectively. For majority of participants (98%, n=1373), CSF Aβ42 and Aβ40 were measured using the Elecsys® CSF electrochemiluminescence immunoassay (Roche Diagnostics) and Roche NeuroToolKit, respectively. For the rest, CSF Aβ42 and Aβ40 were quantified using either Lumipulse G (Fujirebio) or MSD assays. Aβ status (negative/positive) was determined using CSF Aβ42/Aβ40 ratio based on previously described thresholds of 0.080 (Roche)[14] and 0.072 (Lumipulse G) [16]. For MSD CSF Aβ42/Aβ40, the threshold of <0.077 was defined using mixture modeling.

Health and Aging Brain Study – Health Disparities study

Participants

The HABS-HD (Health & Aging Brain Study – Health Disparities, formerly known as the Health & Aging Brain Study among Latino Elders, HABLE), is a longitudinal community-based initiative that examines health disparities related to mild cognitive impairment (MCI) and Alzheimer's disease (AD) in Mexican Americans and African-American compared to non-Hispanic Whites [18]. The methods used in the HABS-HD study have been previously published and are briefly outlined below. The recruitment process follows a community-based participatory research (CBPR) approach, which has proven effective in reaching underserved and minority populations. This approach involves collaboration with local communities through various means, such as organizing community events, seminars, word of mouth, and using different marketing modalities like newspapers, television, and radio. Participants and their healthcare providers are provided with relevant information, including clinical lab work, magnetic resonance imaging (MRI) results, and neuropsychological test outcomes. The study protocol can be conducted in either Spanish or English, accommodating the participants' preferences. The HABS-HD study adheres to institutional review board-approved protocols, and all participants (or their legal representatives)

provided written informed consent. Importantly, all data collected in the HABS-HD study are accessible to the scientific community through the University of North Texas Health Science Center Institute for Translational Research (ITR) website (https://apps.unthsc.edu/itr/). Cognitive diagnoses were determined using an algorithmic approach (decision tree) and later confirmed through a consensus review process. The criteria for each diagnosis were as follows: (i) Normal control (NC): Individuals with no cognitive complaints, a CDR Sum of Boxes score between 0 and 0.5, and cognitive test scores within the normal range (performance higher than the threshold for mild cognitive impairment, which is defined as ≤ 1.5 standard deviations below the normative range) (ii) Mild Cognitive Impairment (MCI): Individuals with cognitive complaints (self-reported or reported by others), a CDR Sum of Boxes score between 0.5 and 2, and at least one cognitive test score falling ≤ 1.5 standard deviations below the normative range and % (iii) Dementia: Individuals with a CDR Sum of Boxes score ≥ 2.5 and at least two cognitive test scores falling 2 standard deviations below the normative ranges.

Biomarker assessments

To prepare the assays, a custom automated StarPlus system from Hamilton Robotics was utilized. Plasma markers such as p-tau181, and neurodegeneration (NfL) were analyzed using the ultrasensitive Simoa (single-molecule array) technology platform HD-X from Quanterix.com, which has been described previously [19]. The coefficients of variation for all these assays were less than or equal to 5%. Amyloid-PET imaging was conducted using FDA-approved [^{18}F]florbetaben (aka Neuraceq) as described previously [18]. Briefly, a dynamic emission scan consisting of four frames, each lasting 5 minutes (totaling 20 minutes), is initiated 90 minutes after the injection of the radiotracer. This is preceded by the acquisition of a low-dose CT scan, which is employed for attenuation correction. Composite ROIs based on FreeSurfer-defined areas (including frontal, anterior/posterior cingulate, lateral parietal, and lateral temporal cortex) were established to generate a summarized cortical ROI. To achieve a global SUVR, normalization to a reference region encompassing the entire cerebellum was carried out. A SUVR value of 1.08 was utilized to establish the threshold for amyloid-PET positivity as previously described [18].

Mayo Clinic Study of Aging (MCSA)

Participants

We assessed 1564 individuals from the Mayo Clinic study of Aging (MCSA), a population-based study of cognitive aging from an age- and sex-stratified random sample of residents of Olmsted County, Minnesota, U.S.A [20]. Participant assessment at the MCSA involves study coordinator interviews with participants, physician examinations, and neuropsychological testing. During the in-clinic examination, participant demographics such as age, sex, and years of education are recorded, alongside their medical history. A blood draw is obtained and APOE ε4 genotyping is conducted. Diagnoses of MCI and dementia are established by a consensus committee that evaluates each participant. Cognitive performance is compared with age-adjusted scores of cognitively unimpaired (CU) individuals from Mayo's Older Americans Normative Studies. Participants with scores around 1.0 standard deviation below the age-specific mean in the general population are considered to have possible cognitive impairment. MCI is diagnosed based on clinical judgment, including patient and informant history, along with published criteria, which include cognitive complaints, abnormal cognitive function for age, essentially normal functional activities, and no dementia [1]. Participants with normal cognitive performance and who do not meet the criteria for MCI or dementia are classified as cognitively unimpaired (CU). The consensus committee making these diagnoses is blinded to plasma P-tau and neuroimaging results, as well as clinical information and diagnoses from previous study visits. The MCSA was approved by the Mayo Clinic and the Olmsted Medical Center Institutional Review Boards. All participants provided written informed consent at the time of enrollment.

Biomarker assessments

Amyloid-PET was assessed using Pittsburgh compound B ([^{11}C]PiB) as described previously [20]. A global cortical [^{11}C]PiB-PET retention ratio was calculated by determining the median uptake across voxels in specific regions of interest (ROIs) such as the prefrontal, orbitofrontal, parietal, temporal, anterior cingulate, and posterior cingulate/precuneus for each participant. This value was then divided by the median uptake across voxels in the cerebellar crus. Blood samples were collected at the clinic following an overnight period of fasting. These blood samples were subjected to centrifugation, and the resulting plasma was divided into smaller portions and stored at a temperature of -80 °C. The assays employed were proprietary and were developed by Lilly Research Laboratories. The samples were diluted at a ratio of 1:2, with 50 µl of the diluted sample utilized for each duplicate measurement. The assays were conducted on a streptavidin-coated plate

featuring small spots and were conducted using the Meso Scale Discovery platform. 1564 individuals had plasma p-tau181, GFAP, NfL and amyloid-PET. 689 also had plasma p-tau217 available assayed using Lilly MSD [23]. 361 (23.1%) also had tau-PET available.

McGill Memory clinic cohort

Participants

We assessed 54 cognitively impaired individuals from the McGill Centre for Studies in Aging who underwent lumbar puncture, clinical evaluation by dementia specialists and had plasma biomarker assessments available. Individuals in the McGill Memory clinic cohort met appropriate use recommendations for AD biomarker investigations [24,25]. Specifically, all individuals in the McGill Memory clinic cohort were either: (1) individuals with dementia, regardless of clinical presentation, who were either of early-onset (below 65 years of age) or (2) individuals with dementia that developed after the age of 65 years, where the prominent clinical aspect is related to any cognitive or behavioral domain other than memory [26]. Individuals who met the standard clinical criteria for probable AD [6] and individuals with non-AD dementias were not included.

Biomarker assessments

CSF samples from the memory clinic cohort were shipped to a commercial laboratory (Athena Diagnostics, Worcester MA) for analyses of Amyloid-β (1-42), total tau (T-tau) and p-tau_{181} using Admark® ELISA kit. Participants in the memory clinic cohort underwent clinical lumbar puncture according to appropriate use criteria for AD as described previously [26]. Plasma biomarker assessments are performed according to the same procedures as the TRIAD cohort (see TRIAD section).

Sant Pau Initiative on Neurodegeneration (SPIN)

Participant information

We assessed 596 individuals with plasma biomarkers and CSF assessments of Aβ42/40 and p-tau181 from the Sant Pau Initiative on Neurodegeneration (SPIN) cohort [27]. All participants were evaluated with a comprehensive neurological assessment, along with a structural brain MRI using a 3T scanner. All participants in the SPIN group undergo a typical one-hour neuropsychological assessment. This assessment evaluates abilities such as remembering spoken information, recalling

visual information, focusing attention, managing cognitive tasks, understanding spatial relationships, visual perception, constructing visual patterns, and language skills. Additionally, the assessment covers neuropsychiatric symptoms, how these symptoms affect daily functioning, and the extent of overall cognitive decline.

Biomarker assessments

Every participant in the SPIN cohort is required to undergo a lumbar puncture in order to obtain cerebrospinal fluid (CSF) samples. These samples are collected and processed in accordance with international recommendations [28], using polypropylene tubes and are stored at a temperature of -80°C until analysis. Key AD biomarkers including CSF Aβ1-42, Aβ1-40, t-tau, and p-tau are regularly measured in all participants using the fully automated platform Lumipulse (Fujirebio-Europe). Cutoffs for CSF amyloid positivity (Aβ1-42/Aβ1-40 ratio < 0.062) and for CSF p-tau positivity (p-tau > 63pg/ml) were established to maximize the agreement between CSF markers and amyloid PET [29]. To ensure the accuracy of our results, our laboratory takes part in the Alzheimer's Association quality control program for CSF biomarkers [28].

Blood samples were collected in 10 ml EDTA-2K tubes and then centrifuged for 10 minutes at 4°C. Plasma was aliquoted into polypropylene tubes and stored at -80ºC until analysis. Plasma p-tau181 and plasma p-tau231 were measured using the in-house Simoa assay developed in University of Gothenburg [30]. Concentration of p-tau217 was obtained using the Simoa-based ALZPath assay [8]. GFAP and NfL were measured using the Simoa Quanterix assay [31,32].

Translational Biomarkers in Aging and Dementia

Participant information

We assessed 319 participants in the prospective Translational Biomarkers of Aging and Dementia (TRIAD)[33] cohort, a longitudinal observational cohort study in Montréal, Québec, Canada. The cohort participants had a detailed clinical assessments from dementia specialists, including the Clinical Dementia Rating (CDR) and Mini-Mental State Examination (MMSE). Cognitively unimpaired (CU) individuals had no objective cognitive impairment and a CDR score of 0. Individuals with MCI had subjective and/or objective cognitive impairment and a CDR score of 0.5 [1]. Individuals with dementia had a CDR score of 1 or 2 [48]. Participants were excluded from

this study if they had systemic conditions which were not adequately controlled through a stable medication regimen. Other exclusion criteria were active substance abuse, recent head trauma, recent major surgery, or MRI/PET safety contraindications. The study was approved by the Montreal Neurological Institute PET working committee and the Douglas Mental Health University Institute Research Ethics Board. Written informed consent was obtained for all participants.

Biomarker assessments

Participants in the TRIAD cohort were evaluated with five plasma biomarkers (p-tau181, p-tau217, p-tau231, GFAP and NfL), as well as amyloid-PET with [^{18}F]AZD4694. Acquisition and processing of PET data was been described previously [34]. Plasma p-tau_{181} and p-tau_{231} were quantified in the Clinical Neurochemistry Laboratory, University of Gothenburg by scientists blinded to participant clinical and PET information, as described previously, and plasma p-tau_{217} was quantified by scientists at Janssen R&D blinded to clinical and PET information [35].

University of California San Diego Shiley-Marcos Alzheimer's Disease Research Center

Participants

Participants were volunteers who were part of the UCSD Shiley-Marcos Alzheimer's Disease Research Center (ADRC). 177 participants took part in yearly assessments and were followed until they passed away. Additionally, they agreed to have their brains examined after death. The research plan underwent a review and gained approval from the human subject review board at the University of California, San Diego (UCSD). All patients or their caregivers provided informed consent as per the regulations of California State law. A comprehensive yearly clinical assessment included input from knowledgeable sources, medical and neurological history, evaluations of mental state, examination of psychiatric symptoms using the Neuropsychiatric Inventory (NPI), assessments of functional capability, a total score for Clinical Dementia Rating (CDR) along with six subdomain scores (referred to as CDR sum of boxes), a structured neurological examination, and a neuropsychological assessment. The neuropsychological assessment used a wide range of cognitive tests, including global cognition assessments like the Mini-Mental State Examination (MMSE) and Dementia Rating Scale (DRS), as well as tests for memory, language, attention, executive function, and visuospatial abilities. At least two tests were

conducted for each cognitive domain. Following each yearly evaluation, participants were given a research diagnosis during a consensus conference. This diagnosis determined an overall evaluation of their cognitive status, categorizing it as cognitively unimpaired (CU), mild cognitive impairment (MCI) diagnosed using standard criteria [1], or dementia [6].

Neuropathological and biomarker assessments

Autopsies were performed using a standardized protocol as described previously [36]. Neuritic plaques, diffuse plaques, and neurofibrillary tangles (NFTs) were detected using either 1% thioflavin-S stains under ultraviolet light with a 440 µm bandpass wavelength excitation filter, or through immunohistochemical staining with antibodies targeting Aβ and PHF1 tau. The density of neuritic plaques was estimated using methodologies recommended by CERAD [37], and the level of NFT pathology was determined according to the Braak stage [38]. Plasma was processed in accordance with the established procedures of the UCSD Shiley-Marcos ADRC. Blood was drawn from a vein in the forearm using EDTA citrate vacutainer tubes. Subsequently, the blood samples were centrifuged at 2000×g for a duration of 10 minutes at a temperature of 4°C using a tabletop centrifuge, all within an hour or less from the time of blood collection. The resulting plasma was separated, divided into 500 µL portions, and placed into cryo-tubes made of polypropylene from VWR or Sarstedt. These samples were then rapidly frozen by snap freezing and stored at a temperature of -80ºC until the time of conducting biomarker analyses. To measure plasma biomarkers, Single molecule array (Simoa) assays from Quanterix were employed. These assays included P-tau181 using the pTau-181 V2 Advantage Kit #103714, P-tau231 (as established by the University of Gothenburg) [39], and NfL (NF-light™ Advantage Kit #103186). The analysis of these biomarkers was carried out at the Clinical Neurochemistry Laboratory situated at the University of Gothenburg, Sweden.

Supplemental Table 1: Representativeness of the study participants

**Disease**	Alzheimer's disease
**Age**	Prevalence of AD increases strongly with age.
**Sex**	AD is more frequently diagnosed in females than in males.
**Race and ethnicity**	Risk of dementia is greater among Black/African American and Hispanic/Latinx individuals than non-Hispanic white individuals. Data is lacking on prevalence of AD as a cause of dementia in different racial and ethnic groups.
**Geography**	This study included cohorts from Canada, France, South Korea, Spain, Sweden, and the United States. The relationship between geography and AD prevalence is poorly understood.
**Other considerations**	Published prevalence estimates of AD pathology used this study were derived from the Amyloid Biomarker Study Group, which may also have limited generalizability, especially in terms of race and ethnicity. Furthermore, because of associations with years of education and dementia risk, we reported level of education of the samples. Most samples were highly educated, which may limit generalizability, though some cohorts such as the Biobank Innovations for chronic Cerebrovascular disease With ALZheimer's disease Study (BICWALZS) predominantly consisted of individuals with less years of education.
**Overall representativeness of the study**	As a whole, the demographics of this study do not reflect the populations at risk for dementia or AD in North America in terms of race and ethnicity. However, some of the cohort studies investigated in this study were much more diverse: the Health & Aging Brain – Health Disparities (HABS-HD) and Biobank Innovations for chronic Cerebrovascular disease With ALZheimer's disease Study (BICWALZS). The HABS-HD was 21% Mexican-American and 47% African-American and the BICWALZS study (based in South Korea) was 100% Asian.

Supplemental Table 2: Comparative characteristics of the different cohorts

Cohort (ref)	No. total	No. CU	No. CI	Setting	Location	Reference standard	Plasma biomarkers assessed	Age, mean (SD)	N (%) female	Education, years (SD)	MMSE, mean (SD)
**ADNI** [4]	1091	383	708	Research	Canada & USA, Multicentre	CSF & Amyloid-PET	p-tau181, NfL	CU: 73.6 (5.8)<br>CI: 72.8 (7.6)	CU: 203 (53%)<br>CI: 303 (42.8%)	CU: 16.5 (2.6)<br>CI: 16.0 (2.7)	CU: 28.9 (1.5)<br>CI: 26.2 (3.4)
**BICWALZS** [7]	727	89	638	Clinical	Multicentre, South Korea	Amyloid-PET	p-tau217, GFAP	CU: 70.2 (7.4)<br>CI: 72.8 (7.6)	CU: 68 (76.4%)<br>CI: 422 (66.1%)	CU: 8.16 (4.6)<br>CI: 8.39 (4.9)	CU: 27 (2.46)<br>CI: 22.6 (5.14)
**BioCogBank** [41]	216	21	195	Clinical	Paris, France	CSF	p-tau181, p-tau231, GFAP, NfL	CU: 64.4 (9.50)<br>CI: 69.9 (8.99)	CU: 14 (66.7%)<br>CI: 114 (58.5%)	CU: 11.2 (1.6)<br>CI: 10.5 (1.9)	CU: 27.2(2.52)<br>CI: 22.1(5.39)
**BIODEGMAR** [10]	210	33	177	Clinical	Barcelona, Spain	CSF	p-tau181, p-tau217, p-tau231, GFAP, NfL	CU: 70.3 (6.3)<br>CI: 72.3 (5.8)	CU: 15 (45.5%)<br>CI: 115 (54.8%)	CU: 10.8 (4.5)<br>CI: 8.5 (4.3)	CU: 28.1 (1.67)<br>CI: 22.5 (5.28)
**BioFINDER-2** [12]	1373	709	664	Clinical	Lund, Sweden	CSF	p-tau181, p-tau217, p-tau231, GFAP, NfL	CU: 63.6 (14.6)<br>CI: 72.2 (7.9)	CU: 375 (52.9)<br>CI: 297 (44.7)	CU: 13.0 (3.5)<br>CI: 12.5 (4.2)	CU: 28.9 (1.2)<br>CI: 24.5 (4.4)
**HABS-HD** [18]	598	440	158	Community	Fort Worth, TX, USA	Amyloid-PET	p-tau181, NfL	CU: 63.4 (7.76)<br>CI: 66.2 (9.46)	CU: 290 (66%)<br>CI: 101 (64%)	CU: 13.7 (4.12)<br>CI: 13.1 (4.54)	CU: 28.5 (1.73)<br>CI: 26.3 (3.66)
**MCSA** [23]	1559	1381	178	Community	Rochester, MI, USA	Amyloid-PET	p-tau181, p-tau217, GFAP, NfL	CU: 69.7 (10.0)<br>CI: 78.5 (8.54)	CU: 652 (47.2%)<br>CI: 63 (35.4%)	CU: 15.0 (2.49)<br>CI: 13.8 (2.53)	CU: 28.6 (1.16)<br>CI: 25.2 (2.28)
**McGill Memory Clinic** [26]	54	0	54	Clinical	Montreal, Canada	CSF	p-tau181, p-tau217, p-tau231, GFAP, NfL	CI: 63.4 (6.52)	CI: 21 (38.8%)	CI: 13.1 (2.63)	CI: 23.6 (3.58)
**SPIN** [27]	596	196	400	Mixed	Barcelona, Spain	CSF	p-tau181, p-tau217, p-tau231, GFAP, NfL	CU: 54 (13)<br>CI: 73 (7)	CU: 131 (67%)<br>CI: 205 (51%)	CU: 15.6 (4.0)<br>CI: 10.6 (5.1)	CU: 29.2 (0.9)<br>CI: 24.6 (4.0)
**TRIAD** [33]	295	157	138	Research	Montreal, Canada	Amyloid-PET	p-tau181, p-tau217, p-tau231, GFAP, NfL	CU: 63.6 (8.2)<br>CI: 67.7 (9.60)	CU: 120 (53.7)<br>CI: 82 (59.4)	CU: 15.6 (3.4)<br>CI: 14.8 (3.74)	CU: 28.9 (1.10)<br>CI: 24.7 (5.58)
**UCSD ADRC** [36]	177	17	160	Research	San Diego, USA	Neuropathology	p-tau181, p-tau231, GFAP, NfL	CU: 88.9 (5.01)<br>CI: 80.0 (7.72)	CU: 12 (70.6%)<br>CI: 110 (68.8%)	CU: 14.9(3.78)<br>CI: 15.3(3.26)	CU: 28.8(1.29)<br>CI: 17.4(7.40)

Supplemental Table 3: Demographic and clinical characteristics of the Alzheimer's disease Neuroimaging Initiative CSF cohort

	CU	CI
**No.**	295	605
**Age, y, mean (SD)**	73.2 (5.99)	72.1 (7.60)
**Female, no. (%)**	159 (53.9%)	263 (43.5%)
**MMSE, mean, (SD)**	28.9 (1.6)	26.5 (3.1)
***APOE ε4 carriers,* %**	85 (28.8%)	316 (52.2%)
**Education, y, mean (SD)**	16.6 (2.56)	16.1 (2.65)
**Amyloid-β positive, %**	103 (34.9%)	379 (62.6%)
**Racial Category**		
**American Indian / Alaskan Native**	1 (0.3%)	1 (0.2%)
**Asian**	4 (1.4%)	10 (1.7%)
**Black**	16 (5.4%)	15 (2.5%)
**Hawaiian / Pacific Islander**	0 (0%)	2 (0.3%)
**More than one**	5 (1.7%)	8 (1.3%)
**White**	269 (91.2%)	567 (93.7%)
**Unknown / not reported**	0 (0%)	2 (0.3%)
**Ethnic category**		
**Hispanic /Latinx**	11 (3.7%)	20 (3.3%)
**Not Hispanic /Latinx**	281 (95.3%)	583 (96.4%)
**Unknown / not reported**	3 (1.0%)	2 (0.3%)

Supplemental Table 4: Demographic and clinical characteristics of the Alzheimer's disease Neuroimaging Initiative amyloid-PET cohort

	CU	CI
**No.**	88	103
**Age, y, Mean (SD)**	74.7 (5.17)	74.2 (7.76)
**Female, no. (%)**	44 (50.0%)	40 (38.8%)
**MMSE, mean (SD)**	28.9 (1.4)	25.2 (4.4)
***APOE ε4 carriers,* %**	24 (27.3%)	49 (47.6%)
**Education, y, Mean (SD)**	16.4 (2.87)	15.6 (3.00)
**Amyloid-β positive, %**	32 (36.4%)	64 (62.1%)
**Racial Category**		
**Asian**	2 (2.3%)	2 (1.9%)
**Black**	7 (8.0%)	5 (4.9%)
**Hawaiian / Pacific Islander**	0 (0%)	0 (0%)
**More than one**	0 (0%)	1 (1.0%)
**White**	79 (89.8%)	94 (91.3%)
**Unknown / not reported**	0 (0%)	1 (1.0%)
**Ethnic Category**		
**Hispanic / Latinx**	4 (4.5%)	5 (4.9%)
**Not Hispanic / Latinx**	84 (95.5%)	98 (95.1%)
**Unknown / not reported**	0 (0%)	0 (0%)

Supplemental Table 5: Demographic and clinical characteristics of the Biobank Innovations for chronic Cerebrovascular disease With ALZheimer's disease Study

	CU	CI
**No.**	89	638
**Age, y, mean (SD)**	70.2 (7.4)	72.8 (7.6)
**Female, no. (%)**	68 (76.4%)	422 (66.1%)
**MMSE, mean (SD)**	27 (2.46)	22.6 (5.14)
***APOE ε4 carriers,* %**	10 (11.2%)	198 (31.0%)
**Education, y, mean (SD)**	8.16 (4.6)	8.39 (4.9)
**Amyloid-β positive, %**	7 (7.9%)	251 (39.3%)
**Racial category**		
**American Indian / Alaskan Native**	0 (0%)	0 (0%)
**Asian**	89 (100%)	638 (100%)
**Black**	0 (0%)	0 (0%)
**Hawaiian / Pacific Islander**	0 (0%)	0 (0%)
**More than one**	0 (0%)	0 (0%)
**White**	0 (0%)	0 (0%)
**Unknown / not reported**	0 (0%)	0 (0%)
**Ethnic category**		
**Hispanic/Latinx**	0 (0%)	0 (0%)
**Not Hispanic/Latinx**	89 (100%)	638 (100%)
**Unknown / not reported**	0 (0%)	0 (0%)

Supplemental Table 6: Demographic and clinical characteristics of BioCogBank cohort study

	CU	CI
**No.**	21	195
**Age, y, Mean(SD)**	64.4(9.50)	69.9(8.99)
**Female, no. (%)**	14 (66.7%)	114 (58.5%)
**MMSE, Mean (SD)**	27.2(2.52)	22.1(5.39)
**Education, y, mean (SD)**	11.2 (1.6)	10.5 (1.9)
***APOE ε4 carriers,* %**	6 (28.6%)	88 (45.1%)
**Amyloid-β positive, %**	0 (0%)	129 (66.2%)

Race and ethnicity data was not reported in the BioCogBank cohort.

Supplemental Table 7: Demographic and clinical characteristics of BioDEGMAR study

	CU	CI
**No.**	33	177
**Age, y, mean (SD)**	70.3 (6.34)	72.6 (5.68)
**Female, no. (%)**	15 (45.5)	100 (56.5)
**Education, y, mean (SD)**	10.8 (4.49)	8.03 (4.20)
***APOE ε4 carriers,* %**	7 (21.9)	81 (50.3)
**MMSE, mean (SD)**	28.1 (1.67)	21.4 (5.06)
**Racial category**		
**American Indian / Alaskan Native**	0 (0)	0 (0)
**Asian**	0 (0)	1 (0.5)
**Black**	0 (0)	0 (0)
**Hawaiian / Pacific Islander**	0 (0)	0 (0)
**More than one / Other**	0 (0)	1 (0.5)
**White**	33 (100)	175 (99)
**Unknown / not reported**	0 (0)	0 (0)

Supplemental Table 8: Demographic and clinical characteristics of BioFINDER-2 study

	CU	CI
**No.**	709	664
**Age, y, mean (SD)**	63.6 (14.6)	72.2 (7.9)
**Female, no. (%)**	375 (52.9)	297 (44.7)
**Education, y, mean (SD)** [a]	13.0 (3.5)	12.5 (4.2)
***APOE ε4 carriers,* %** [a]	292 (41.2)	362 (54.5)
**MMSE, mean (SD)** [a]	28.9 (1.2)	24.5 (4.4)
**Amyloid-β positive, N, (%)** [b]	172 (24.3)	444 (66.9)

[a] Education was missing for 3 CU and 26 CI; *APOE* data was missing for 92 CU and 3 CI; MMSE was missing for 1 CI. [b] Amyloid positivity was determined using CSF Aβ42/Aβ40.
Self-reported race and ethnicity data are not collected in the Swedish BioFINDER-2 study.

Supplemental Table 9: Demographic and clinical characteristics of Health & Aging Brain study – Health Disparities (HABS-HD) study

	CU	CI
**No.**	440	158
**Age, y, mean (SD)**	63.4 (7.76)	66.2 (9.46)
**Female, no. (%)**	290 (66%)	101 (64%)
**Education, y, mean (SD)**	13.7 (4.12)	13.1(4.54)
**MMSE, mean (SD)**	28.5 (1.73)	26.3 (3.66)
**Amyloid-β positive (%)**	30 (6.8%)	25 (15%)
**Racial category**		
**American Indian / Alaskan Native**	2 (0.45%)	1 (0.63%)
**Asian**	1 (0.27%)	0 (%)
**Black**	204 (46%)	73 (46.2%)
**Hawaiian / Pacific Islander**	1 (0.27%)	1 (0.63%)
**More than one**	9 (2%)	8 (5%)
**White**	223 (%)	75 (47.4%)
**Unknown / not reported**	0 (0%)	0 (0%)
**Ethnic category**		
**Hispanic/Latinx**	86 (19.5%)	41 (%)
**Not Hispanic/Latinx**	354 (80.5%)	117 (%)
**Unknown / not reported**	0 (0%)	0 (0%)

APOE data was not available.

Supplemental Table 10: Demographic and clinical characteristics of Mayo Clinic Study of Aging (MCSA)

	CU	CI
**No.**	1381	178
**Age, y, Mean (SD)**	69.7 (10.0)	78.5 (8.54)
**Female, no. (%)**	652 (47.2%)	63 (35.4%)
**MMSE, Mean (SD)**	28.6 (1.16)	25.2 (2.28)
***APOE ε4 carriers,* %**	366 (26.5%)	64 (36.0%)
**Education, y, mean (SD)**	15.0 (2.49)	13.8 (2.53)
**Amyloid-β positive, %**	366 (26.5%)	103 (57.9%)
**Racial Category**		
**American Indian / Alaskan Native**	0 (0%)	0 (0%)
**Asian**	5 (0.36%)	3 (0%)
**Black**	6 (0.43%)	0 (0%)
**Hawaiian / Pacific Islander**	0 (0%)	0 (0%)
**More than one**	12 (0.9%)	3 (1.7%)
**White**	1352 (97.9%)	171 (96.1%)
**Unknown/Not reported**	6 (0.4%)	1 (0.6%)
**Ethnic category**		
**Hispanic / Latinx**	4 (0.3%)	2 (1.1%)
**Not Hispanic / Latinx**	1369 (99.1%)	174 (97.8%)
**Unknown / not reported**	8 (0.6%)	2 (1.1%)

Supplemental Table 11: Demographic and clinical characteristics of McGill Memory Clinic

	CI
**No.**	54
**Age, y, mean (SD)**	63.4 (6.52)
**Female, no. (%)**	21 (38.8%)
**Education, y, mean (SD)**	13.1 (2.63)
***APOE ε4 carriers,* %**	28 (51.8%)
**MMSE, mean (SD)**	23.6 (3.58)
**Amyloid-β positive, %**	26 (48.1%)
**Racial category**	
**American Indian / Alaskan Native**	0 (0%)
**Asian**	1 (1.8%)
**Black**	0 (0%)
**Hawaiian / Pacific Islander**	0 (0%)
**More than one**	1 (1.8%)
**White**	52 (96.3%)
**Unknown / not reported**	0 (0%)
**Ethnic category**	
**Hispanic/Latinx**	0 (0%)
**Not Hispanic/Latinx**	54 (96.4)
**Unknown / not reported**	0 (0%)

Supplemental Table 12: Demographic and clinical characteristics of the Sant Pau Initiative on Neurodegeneration (SPIN) cohort study

	CU	CI
**No.**	196	400
**Age, y, mean (SD)**	54 (13)	73 (7)
**Female, no. (%)**	131 (67)	205 (51)
**Education, y, mean (SD)**	15.6 (4.0)	10.6 (5.1)
***APOE ε4 carriers,* %**	46 (23%)	125 (32%)
**MMSE, mean (SD)**	29.2 (0.9)	24.6 (4.0)
**Amyloid-β positive, %**	14 (7.1)	188 (47)

Race and ethnicity data are not collected in the SPIN and hence are not reported here.

Supplemental Table 13: Demographic and clinical characteristics of Translational Research in Aging and Dementia (TRIAD) cohort

	CU	CI
**No.**	157	138
**Age, y, mean (SD)**	63.6 (8.2)	67.7 (9.60)
**Female, no. (%)**	120 (53.7)	82 (59.4)
**Education, y, mean (SD)**	15.6 (3.4)	14.8 (3.74)
***APOE ε4 carriers*, %**	49 (31%)	64 (46.4)
**MMSE, mean (SD)**	28.9 (1.1)	24.7 (5.58)
**Amyloid-β positive, %**	38 (24%)	87 (63%)
**Racial category**		
**American Indian / Alaskan Native**	0 (0%)	0 (0%)
**Asian**	0 (0%)	4 (2.8%)
**Black**	2 (1.3%)	1 (0.7%)
**Hawaiian / Pacific Islander**	0 (0%)	0 (0%)
**More than one**	0 (0%)	0 (0%)
**White**	141 (89.8%)	128 (92.3%)
**Unknown / not reported**	14 (8.9%)	5 (3.6%)
**Ethnic category**		
**Hispanic/Latinx**	2 (1.3%)	0 (%)
**Not Hispanic/Latinx**	141 (89.8%)	134 (97.1%)
**Unknown / not reported**	14 (8.9%)	4 (2.8%)

Supplemental Table 14: Demographic and clinical characteristics of University of California San Diego Alzheimer's disease Research Centre (UCSD-ADRC) study

	CU	CI
**No.**	17	160
**Age, y, mean (SD)**	88.9 (5.01)	80.0 (7.72)
**Female, no. (%)**	12 (70.6%)	110 (68.8%)
**Education, y, mean (SD)**	14.9(3.78)	15.3(3.26)
APOE ε4 carriers, %	NA	NA
**MMSE, mean (SD)**	28.8(1.29)	17.4(7.40)
**Reagan**		
High AD	1 (5.9%)	96 (60.0%)
Mod AD	3 (17.6%)	32 (20.0%)
Low AD	13 (76.5%)	32 (20.0%)
**Racial category**		
**American Indian / Alaskan Native**	0 (0%)	0 (0%)
**Asian**	0 (0%)	0 (0%)
**Black**	0 (0%)	2 (1.25%)
**Hawaiian / Pacific Islander**	0 (0%)	0 (0%)
**Other / More than one**	0 (0%)	2 (1.25%)
**White**	17 (100%)	156 (97.5%)
**Unknown / not reported**	0 (0%)	0 (0%)
**Ethnic category**		
**Hispanic/Latinx**	4 (23.5%)	38 (23.8%)
**Not Hispanic/Latinx**	13 (76.5%)	122 (76.2%)
**Unknown / not reported**	0 (0%)	0 (0%)

Supplemental Table 15: Self-reported race data for the entire sample.

	Cognitively unimpaired	Cognitively impaired
**Racial category**		
**Asian**	101 (3%)	658 (18.8%)
**Black**	235 (7%)	96 (2.7%)
**More than one / Other**	24 (0.7%)	30 (0.85%)
**White**	2097 (61.8%)	1418 (40.5%)
**Unknown / not reported**	946 (27.8%)	1268 (36.2%)

Race and ethnicity were reported by the participant. Race and Ethnicity data were not collected in the Swedish BioFINDER-2 study or the Sant Pau Initiative on Neurodegeneration study.

Supplemental Table 16: Positive and Negative Predictive values of plasma biomarkers for amyloid-β pathology in individuals with MCI.

	p-tau181		p-tau217		p-tau231		GFAP		NfL	
**Age**	PPV (95%CI)	NPV (95%CI)	PPV (95%CI)	NPV (95%CI)	PPV (95%CI)	NPV (95%CI)	PPV (95%CI)	NPV (95%CI)	PPV (95%CI)	NPV (95%CI)
**50-54**	51.4% (44.7-56.9)	75.9% (74.4-77.2)	68.4% (65.5-71.4)	94.4% (93.3-95.4)	57.1% (51.1-62.4)	82.2% (80.3-83.8)	59.8% (53.3-65.9)	82.5% (80.7-84.4)	32.1% (28-36.6)	70.9% (69.1-72.7)
**55-59**	57% (50.4-62.4)	71.5% (69.8-72.9)	73.1% (70.5-75.9)	93% (91.7-94.3)	62.6% (56.7-67.6)	78.6% (76.5-80.4)	65.1% (58.9-70.8)	79% (76.9-81.2)	37.3% (32.8-42)	66% (64-67.9)
**60-64**	62.2% (55.7-67.2)	66.9% (65.1-68.5)	77.1% (74.7-79.6)	91.5% (89.9-93)	67.4% (61.9-72.1)	74.8% (72.4-76.8)	69.8% (64-75)	75.2% (72.9-77.7)	42.4% (37.7-47.3)	61% (59-63.1)
**65-69**	67.3% (61.2-72)	61.8% (59.8-63.5)	80.9% (78.7-83)	89.6% (87.7-91.4)	72.2% (67-76.4)	70.3% (67.7-72.6)	74.3% (69-79)	70.8% (68.3-73.6)	47.9% (43.1-52.9)	55.6% (53.4-57.7)
**70-74**	72% (66.3-76.2)	56.5% (54.4-58.2)	84.1% (82.2-85.9)	87.3% (85.1-89.5)	76.4% (71.7-80.1)	65.5% (62.7-68)	78.3% (73.5-82.4)	66% (63.3-69.1)	53.5% (48.5-58.4)	50.1% (47.9-52.2)
**75-79**	77.3% (72.3-80.9)	49.4% (47.4-51.2)	87.5% (86-89)	83.9% (81.2-86.6)	81.1% (77.1-84.3)	58.9% (55.9-61.5)	82.7% (78.6-86.1)	59.4% (56.5-62.7)	60.4% (55.6-65)	43.1% (41-45.2)
**80-84**	80.8% (76.3-84)	44.2% (42.2-46)	89.6% (88.3-90.9)	80.8% (77.8-83.9)	84.1% (80.6-86.8)	53.7% (50.7-56.5)	85.5% (82-88.5)	54.3% (51.3-57.7)	65.3% (60.7-69.6)	38% (36-40.1)
**85-89**	83.4% (79.3-86.2)	39.9% (38-41.7)	91.1% (90-92.2)	77.9% (74.6-81.4)	86.3% (83.2-88.7)	49.3% (46.3-52.1)	87.6% (84.4-90.1)	49.9% (46.9-53.4)	69.1% (64.8-73.2)	34% (32.1-35.9)
**90-95**	85.8% (82.2-88.3)	35.5% (33.7-37.2)	92.5% (91.6-93.5)	74.6% (70.9-78.4)	88.4% (85.6-90.5)	44.7% (41.7-47.4)	89.5% (86.7-91.7)	45.3% (42.3-48.7)	73% (68.9-76.7)	29.9% (28.1-31.7)

Supplemental Table 17: Positive and Negative Predictive values of plasma biomarkers for amyloid-β pathology in individuals with MCI who are APOEε4 noncarriers

	p-tau181		p-tau217		p-tau231		GFAP		NfL	
**Age**	PPV (95%CI)	NPV (95%CI)	PPV (95%CI)	NPV (95%CI)	PPV (95%CI)	NPV (95%CI)	PPV (95%CI)	NPV (95%CI)	PPV (95%CI)	NPV (95%CI)
**50-54**	33.6% (27.9-38.7)	86.8% (85.8-87.6)	50.9% (47.6-54.5)	97.2% (96.7-97.7)	38.9% (33.3-44.2)	90.6% (89.5-91.5)	41.5% (35.3-48)	90.8% (89.8-91.9)	18.4% (15.7-21.6)	83.6% (82.4-84.8)
**55-59**	38.1% (32.1-43.5)	84.4% (83.2-85.3)	55.8% (52.6-59.3)	96.6% (96-97.3)	43.7% (37.8-49.2)	88.8% (87.5-89.8)	46.5% (40-52.9)	89% (87.8-90.3)	21.6% (18.5-25.2)	80.7% (79.3-82)
**60-64**	42.4% (36.1-47.9)	81.9% (80.6-82.9)	60.2% (57-63.6)	96% (95.2-96.8)	48.1% (42.1-53.6)	86.9% (85.4-88.1)	50.9% (44.3-57.3)	87.1% (85.7-88.6)	24.8% (21.3-28.7)	77.8% (76.2-79.2)
**65-69**	47.7% (41.1-53.2)	78.5% (77.1-79.7)	65.2% (62.1-68.3)	95.1% (94.2-96)	53.5% (47.4-58.9)	84.2% (82.6-85.7)	56.2% (49.7-62.5)	84.6% (82.9-86.3)	29% (25.1-33.2)	73.8% (72.1-75.5)
**70-74**	54.9% (48.2-60.2)	73.3% (71.6-74.6)	71.4% (68.6-74.2)	93.6% (92.4-94.8)	60.5% (54.5-65.6)	80% (78-81.8)	63.1% (56.8-68.9)	80.4% (78.5-82.5)	35.2% (30.9-39.9)	67.9% (66-69.8)
**75-79**	61.2% (54.7-66.3)	67.8% (66-69.4)	76.4% (73.9-78.9)	91.8% (90.3-93.3)	66.5% (60.9-71.2)	75.6% (73.2-77.6)	68.9% (63-74.2)	76% (73.7-78.4)	41.4% (36.7-46.3)	62% (60-64)
**80-84**	66.9% (60.7-71.6)	62.3% (60.3-63.9)	80.5% (78.4-82.7)	89.8% (87.9-91.6)	71.8% (66.6-76)	70.7% (68.1-73)	74% (68.6-78.6)	71.2% (68.7-74)	47.4% (42.6-52.4)	56.1% (53.9-58.2)
**85-89**	72% (66.3-76.2)	56.5% (54.4-58.2)	84% (82.2-85.9)	87.3% (85.1-89.5)	76.4% (71.7-80.1)	65.5% (62.7-68)	78.3% (73.5-82.4)	66% (63.3-69.1)	53.5% (48.5-58.4)	50.1% (47.9-52.2)
**90-95**	76.6% (71.5-80.3)	50.4% (48.4-52.2)	87% (85.5-88.6)	84.4% (81.8-87)	80.5% (76.3-83.7)	59.8% (56.9-62.5)	82.1% (77.9-85.6)	60.4% (57.5-63.7)	59.4% (54.6-64.1)	44.1% (41.9-46.2)

Supplemental Table 18: Positive and Negative Predictive values of plasma biomarkers for amyloid-β pathology in individuals with MCI who are APOEε4 carriers

	p-tau181		p-tau217		p-tau231		GFAP		NfL	
**Age**	PPV (95%CI)	NPV (95%CI)	PPV (95%CI)	NPV (95%CI)	PPV (95%CI)	NPV (95%CI)	PPV (95%CI)	NPV (95%CI)	PPV (95%CI)	NPV (95%CI)
**50-54**	52.6% (45.9-58)	75% (73.4-76.3)	69.4% (66.6-72.4)	94.1% (93-95.2)	58.2% (52.2-63.5)	81.5% (79.6-83.1)	60.9% (54.5-66.9)	81.8% (80-83.8)	33.1% (28.9-37.7)	69.9% (68.1-71.7)
**55-59**	60.2% (53.7-65.3)	68.8% (67-70.3)	75.6% (73.1-78.2)	92.1% (90.7-93.6)	65.6% (59.9-70.4)	76.3% (74.1-78.3)	68% (62-73.4)	76.8% (74.6-79.1)	40.3% (35.7-45.2)	63% (61-65)
**60-64**	69.5% (63.6-74)	59.4% (57.4-61.1)	82.4% (80.4-84.3)	88.6% (86.6-90.6)	74.1% (69.2-78.2)	68.2% (65.5-70.5)	76.2% (71.1-80.6)	68.7% (66-71.6)	50.5% (45.5-55.4)	53.1% (50.9-55.2)
**65-69**	75.8% (70.6-79.7)	51.5% (49.4-53.3)	86.6% (85-88.1)	84.9% (82.4-87.5)	79.8% (75.6-83.1)	60.8% (57.9-63.5)	81.5% (77.2-85.1)	61.4% (58.5-64.6)	58.4% (53.6-63.1)	45.1% (42.9-47.2)
**70-74**	82.7% (78.6-85.7)	41% (39-42.8)	90.8% (89.6-91.9)	78.7% (75.4-82.1)	85.8% (82.5-88.3)	50.4% (47.4-53.2)	87.1% (83.8-89.7)	51% (48.1-54.5)	68.2% (63.8-72.3)	35% (33-37)
**75-79**	87.5% (84.3-89.8)	32.2% (30.4-33.8)	93.5% (92.7-94.3)	71.6% (67.7-75.8)	89.8% (87.4-91.7)	41% (38.1-43.7)	90.8% (88.4-92.8)	41.6% (38.7-45)	75.8% (72.1-79.3)	26.8% (25.2-28.6)
**80-84**	91.3% (89-92.9)	24% (22.6-25.4)	95.6% (95-96.1)	62.7% (58.3-67.6)	93% (91.2-94.3)	31.7% (29.1-34.1)	93.7% (91.9-95)	32.2% (29.6-35.3)	82.5% (79.4-85.2)	19.7% (18.3-21.1)
**85-89**	94.3% (92.7-95.4)	16.8% (15.7-17.8)	97.1% (96.7-97.5)	51.7% (47.1-57.1)	95.4% (94.2-96.3)	22.8% (20.7-24.8)	95.9% (94.7-96.8)	23.2% (21.2-25.8)	88.1% (85.8-90)	13.5% (12.5-14.5)
**90-95**	95.7% (94.4-96.5)	13% (12.1-13.9)	97.9% (97.6-98.1)	44.4% (39.9-49.7)	96.5% (95.6-97.2)	18% (16.3-19.7)	96.9% (96-97.6)	18.4% (16.6-20.5)	90.9% (89.1-92.4)	10.4% (9.6-11.2)

Supplemental Table 19: Positive and Negative Predictive values of plasma biomarkers for amyloid-β pathology in individuals with probable AD dementia

	p-tau181		p-tau217		p-tau231		GFAP		NfL	
**Age**	PPV (95%CI)	NPV (95%CI)	PPV (95%CI)	NPV (95%CI)	PPV (95%CI)	NPV (95%CI)	PPV (95%CI)	NPV (95%CI)	PPV (95%CI)	NPV (95%CI)
**50-54**	95.6% (94.4-96.5)	13.2% (12.2-14)	97.5% (96.5-98.1)	44.6% (40.1-50)	96.5% (95.6-97.2)	18.2% (16.4-19.9)	96.9% (96-97.6)	18.5% (16.8-20.7)	90.8% (89-92.3)	10.5% (9.7-11.3)
**55-59**	95.5% (94.2-96.3)	13.7% (12.7-14.5)	97.4% (96.3-98)	45.7% (41.2-51.1)	96.4% (95.4-97.1)	18.8% (17-20.6)	96.7% (95.8-97.5)	19.2% (17.4-21.4)	90.4% (88.5-92)	10.9% (10.1-11.8)
**60-64**	95.2% (93.9-96.1)	14.3% (13.3-15.2)	97.3% (96.1-97.9)	47% (42.4-52.4)	96.2% (95.2-96.9)	19.6% (17.8-21.5)	96.6% (95.6-97.3)	20% (18.2-22.3)	89.9% (88-91.6)	11.4% (10.6-12.3)
**65-69**	94.8% (93.3-95.8)	15.5% (14.5-16.5)	97% (95.7-97.7)	49.5% (44.8-54.8)	95.8% (94.7-96.6)	21.2% (19.3-23.2)	96.2% (95.1-97.1)	21.6% (19.7-24.1)	89% (86.9-90.8)	12.5% (11.6-13.4)
**70-74**	94.3% (92.7-95.4)	16.8% (15.7-17.8)	96.7% (95.4-97.5)	51.7% (47.1-57.1)	95.4% (94.2-96.3)	22.8% (20.7-24.8)	95.9% (94.7-96.8)	23.2% (21.2-25.8)	88.1% (85.8-90)	13.5% (12.5-14.5)
**75-79**	93.8% (92.1-95)	18% (16.8-19.1)	96.5% (95-97.3)	53.9% (49.3-59.2)	95% (93.7-96)	24.3% (22.2-26.5)	95.5% (94.2-96.5)	24.8% (22.6-27.5)	87.1% (84.8-89.2)	14.5% (13.5-15.6)
**80-84**	93.3% (91.5-94.6)	19.2% (18-20.4)	96.2% (94.6-97.1)	55.9% (51.3-61.1)	94.6% (93.2-95.6)	25.9% (23.6-28.1)	95.2% (93.8-96.2)	26.3% (24.1-29.1)	86.2% (83.7-88.4)	15.6% (14.5-16.7)
**85-89**	92.6% (90.5-94)	21% (19.7-22.3)	95.7% (93.9-96.7)	58.6% (54.1-63.7)	94% (92.5-95.1)	28.1% (25.7-30.4)	94.6% (93.1-95.8)	28.6% (26.2-31.5)	84.8% (82.1-87.2)	17.1% (15.9-18.4)
**90-95**	91.8% (89.6-93.3)	22.8% (21.4-24.1)	95.3% (93.3-96.4)	61.2% (56.7-66.1)	93.4% (91.7-94.6)	30.2% (27.8-32.7)	94.1% (92.4-95.3)	30.8% (28.3-33.8)	83.4% (80.5-86)	18.6% (17.4-20)

Supplemental Table 20: Positive and Negative Predictive values of plasma biomarkers for amyloid-β pathology in individuals with probable AD dementia who are APOEε4 noncarriers

	p-tau181		p-tau217		p-tau231		GFAP		NfL	
**Age**	PPV (95%CI)	NPV (95%CI)	PPV (95%CI)	NPV (95%CI)	PPV (95%CI)	NPV (95%CI)	PPV (95%CI)	NPV (95%CI)	PPV (95%CI)	NPV (95%CI)
**50-54**	89.7% (87-91.6)	27.6% (25.9-29)	94.7% (94-95.4)	66.9% (62.7-71.5)	91.7% (89.6-93.2)	35.8% (33-38.4)	92.5% (90.4-94.1)	36.3% (33.6-39.6)	79.6% (76.3-82.7)	22.7% (21.3-24.3)
**55-59**	90% (87.3-91.8)	27% (25.4-28.4)	94.9% (94.2-95.5)	66.3% (62-70.9)	91.9% (89.9-93.4)	35.1% (32.4-37.7)	92.7% (90.7-94.3)	35.7% (33-38.9)	80.1% (76.8-83.1)	22.2% (20.8-23.8)
**60-64**	90.5% (88-92.3)	25.8% (24.3-27.2)	95.1% (94.5-95.8)	64.9% (60.6-69.6)	92.3% (90.4-93.8)	33.8% (31.1-36.3)	93.1% (91.2-94.6)	34.3% (31.6-37.5)	81.1% (77.8-83.9)	21.2% (19.8-22.7)
**65-69**	90.7% (88.2-92.4)	25.5% (24-26.9)	95.2% (94.6-95.8)	64.6% (60.2-69.3)	92.4% (90.6-93.9)	33.4% (30.8-36)	93.2% (91.3-94.7)	34% (31.3-37.1)	81.3% (78.1-84.1)	21% (19.6-22.4)
**70-74**	90.8% (88.3-92.5)	25.2% (23.7-26.6)	95.3% (94.7-95.9)	64.2% (59.9-69)	92.5% (90.7-93.9)	33.1% (30.4-35.6)	93.3% (91.4-94.7)	33.6% (31-36.8)	81.5% (78.4-84.3)	20.7% (19.3-22.2)
**75-79**	90.9% (88.5-92.6)	24.9% (23.4-26.3)	95.4% (94.7-96)	63.8% (59.5-68.6)	92.7% (90.8-94)	32.7% (30.1-35.2)	93.4% (91.6-94.8)	33.3% (30.6-36.4)	81.8% (78.6-84.5)	20.4% (19.1-21.9)
**80-84**	91.1% (88.6-92.7)	24.6% (23.1-26)	95.4% (94.8-96)	63.5% (59.1-68.3)	92.8% (91-94.1)	32.4% (29.8-34.9)	93.5% (91.7-94.9)	32.9% (30.3-36)	82% (78.9-84.7)	20.2% (18.8-21.6)
**85-89**	91.3% (89-92.9)	24% (22.6-25.4)	95.6% (95-96.1)	62.7% (58.3-67.6)	93% (91.2-94.3)	31.7% (29.1-34.1)	93.7% (91.9-95)	32.2% (29.6-35.3)	82.5% (79.4-85.2)	19.7% (18.3-21.1)
**90-95**	91.8% (89.6-93.3)	22.8% (21.4-24.1)	95.8% (95.3-96.4)	61.2% (56.7-66.1)	93.4% (91.7-94.6)	30.2% (27.8-32.7)	94.1% (92.4-95.3)	30.8% (28.3-33.8)	83.4% (80.5-86)	18.6% (17.4-20)

Supplemental Table 21: Positive and Negative Predictive values of plasma biomarkers for amyloid-β pathology in individuals with probable AD dementia who are APOEε4 carriers

	**p-tau181**		**p-tau217**		**p-tau231**		**GFAP**		**NfL**	
**Age**	PPV (95%CI)	NPV (95%CI)	PPV (95%CI)	NPV (95%CI)	PPV (95%CI)	NPV (95%CI)	PPV (95%CI)	NPV (95%CI)	PPV (95%CI)	NPV (95%CI)
**50-54**	93.3% (91.5-94.6)	19.2% (18-20.4)	96.6% (96.2-97.1)	55.9% (51.3-61.1)	94.6% (93.2-95.6)	25.9% (23.6-28.1)	95.2% (93.8-96.2)	26.3% (24.1-29.1)	86.2% (83.7-88.4)	15.6% (14.5-16.7)
**55-59**	93.4% (91.6-94.7)	18.9% (17.7-20.1)	96.7% (96.2-97.1)	55.4% (50.8-60.6)	94.7% (93.4-95.7)	25.5% (23.2-27.7)	95.3% (93.9-96.3)	25.9% (23.7-28.7)	86.4% (84-88.6)	15.3% (14.2-16.5)
**60-64**	93.6% (91.8-94.8)	18.6% (17.4-19.7)	96.8% (96.3-97.2)	54.9% (50.3-60.1)	94.8% (93.5-95.8)	25.1% (22.9-27.3)	95.3% (94-96.4)	25.6% (23.3-28.3)	86.7% (84.2-88.8)	15% (14-16.2)
**65-69**	93.7% (91.9-94.9)	18.3% (17.1-19.4)	96.8% (96.4-97.2)	54.4% (49.8-59.6)	94.9% (93.6-95.9)	24.7% (22.5-26.9)	95.4% (94.1-96.4)	25.2% (23-27.9)	86.9% (84.5-89)	14.8% (13.7-15.9)
**70-74**	93.8% (92.1-95)	18% (16.8-19.1)	96.9% (96.5-97.3)	53.9% (49.3-59.2)	95% (93.7-96)	24.3% (22.2-26.5)	95.5% (94.2-96.5)	24.8% (22.6-27.5)	87.1% (84.8-89.2)	14.5% (13.5-15.6)
**75-79**	94.3% (92.7-95.4)	16.8% (15.7-17.8)	97.1% (96.7-97.5)	51.7% (47.1-57.1)	95.4% (94.2-96.3)	22.8% (20.7-24.8)	95.9% (94.7-96.8)	23.2% (21.2-25.8)	88.1% (85.8-90)	13.5% (12.5-14.5)
**80-84**	94.8% (93.3-95.8)	15.5% (14.5-16.5)	97.4% (97-97.7)	49.5% (44.8-54.8)	95.8% (94.7-96.6)	21.2% (19.3-23.2)	96.2% (95.1-97.1)	21.6% (19.7-24.1)	89% (86.9-90.8)	12.5% (11.6-13.4)
**85-89**	95% (93.6-95.9)	14.9% (13.9-15.9)	97.5% (97.2-97.8)	48.2% (43.7-53.6)	96% (94.9-96.8)	20.4% (18.5-22.3)	96.4% (95.4-97.2)	20.8% (18.9-23.2)	89.5% (87.5-91.2)	11.9% (11.1-12.9)
**90-95**	95.2% (93.9-96.1)	14.3% (13.3-15.2)	97.6% (97.3-97.9)	47% (42.4-52.4)	96.2% (95.2-96.9)	19.6% (17.8-21.5)	96.6% (95.6-97.3)	20% (18.2-22.3)	89.9% (88-91.6)	11.4% (10.6-12.3)

Supplemental Table 22: Positive and Negative Predictive values of plasma biomarkers for amyloid-β pathology in individuals with frontotemporal dementia

	p-tau181		p-tau217		p-tau231		GFAP		NfL	
**Age**	PPV (95%CI)	NPV (95%CI)	PPV (95%CI)	NPV (95%CI)	PPV (95%CI)	NPV (95%CI)	PPV (95%CI)	NPV (95%CI)	PPV (95%CI)	NPV (95%CI)
**50-54**	15.7% (12.4-18.8)	94.7% (94.3-95.1)	27.6% (25-30.5)	99% (98.8-99.2)	18.9% (15.5-22.6)	96.3% (95.9-96.7)	20.7% (16.7-25.3)	96.4% (96-96.9)	7.7% (6.4-9.2)	93.3% (92.7-93.8)
**55-59**	17.7% (14.1-21.1)	93.9% (93.5-94.3)	30.5% (27.8-33.7)	98.8% (98.6-99)	21.3% (17.5-25.2)	95.8% (95.3-96.2)	23.2% (18.8-28.1)	95.9% (95.4-96.4)	8.8% (7.3-10.5)	92.3% (91.7-92.9)
**60-64**	19.6% (15.7-23.3)	93.2% (92.6-93.6)	33.3% (30.5-36.6)	98.6% (98.4-98.9)	23.5% (19.4-27.7)	95.2% (94.7-95.7)	25.6% (20.9-30.8)	95.3% (94.8-95.9)	9.8% (8.2-11.7)	91.3% (90.6-92)
**65-69**	25.2% (20.5-29.6)	90.8% (90.1-91.4)	40.8% (37.7-44.3)	98.1% (97.8-98.5)	29.7% (24.9-34.6)	93.5% (92.8-94.2)	32.1% (26.7-38)	93.7% (92.9-94.5)	13.1% (11-15.5)	88.4% (87.5-89.3)
**70-74**	30.3% (25-35.2)	88.4% (87.6-89.2)	47.2% (43.9-50.7)	97.6% (97.1-98.1)	35.4% (30.1-40.6)	91.8% (90.8-92.6)	38% (32-44.3)	92% (91.1-92.9)	16.3% (13.8-19.2)	85.5% (84.4-86.6)
**75-79**	35.1% (29.3-40.3)	86% (85-86.9)	52.6% (49.3-56.2)	97% (96.4-97.6)	40.5% (34.8-46)	90% (88.9-91)	43.2% (36.9-49.7)	90.2% (89.1-91.4)	19.5% (16.6-22.8)	82.6% (81.4-83.8)
**80-84**	39.6% (33.4-45)	83.5% (82.4-84.5)	57.3% (54.1-60.8)	96.4% (95.7-97.1)	45.2% (39.3-50.7)	88.1% (86.8-89.3)	48% (41.5-54.5)	88.4% (87.1-89.7)	22.7% (19.4-26.4)	79.7% (78.3-81.1)
**85-89**	43.8% (37.4-49.3)	81% (79.7-82.1)	61.5% (58.3-64.8)	95.8% (95-96.6)	49.5% (43.5-55)	86.2% (84.7-87.5)	52.3% (45.7-58.7)	86.5% (85-88)	25.8% (22.3-29.8)	76.8% (75.2-78.3)
**90-95**	47.7% (41.1-53.2)	78.5% (77.1-79.7)	65.2% (62.1-68.3)	95.1% (94.2-96)	53.5% (47.4-58.9)	84.2% (82.6-85.7)	56.2% (49.7-62.5)	84.6% (82.9-86.3)	29% (25.1-33.2)	73.8% (72.1-75.5)

Supplemental Table 23: Positive and Negative Predictive values of plasma biomarkers for amyloid-β pathology in individuals with Vascular dementia

	p-tau181		p-tau217		p-tau231		GFAP		NfL	
**Age**	PPV (95%CI)	NPV (95%CI)	PPV (95%CI)	NPV (95%CI)	PPV (95%CI)	NPV (95%CI)	PPV (95%CI)	NPV (95%CI)	PPV (95%CI)	NPV (95%CI)
**50-54**	28.7% (23.5-33.4)	89.2% (88.4-89.9)	45.2% (41.9-48.7)	97.8% (97.3-98.2)	33.6% (28.4-38.7)	92.4% (91.5-93.1)	36.1% (30.3-42.3)	92.6% (91.7-93.5)	15.2% (12.9-18)	86.5% (85.5-87.5)
**55-59**	32% (26.5-37)	87.6% (86.7-88.4)	49.1% (45.8-52.7)	97.4% (96.9-97.9)	37.2% (31.7-42.5)	91.2% (90.2-92.1)	39.8% (33.7-46.2)	91.4% (90.4-92.4)	17.4% (14.7-20.4)	84.6% (83.4-85.7)
**60-64**	35.1% (29.3-40.3)	86% (85-86.9)	52.6% (49.3-56.2)	97% (96.4-97.6)	40.5% (34.8-46)	90% (88.9-91)	43.2% (36.9-49.7)	90.2% (89.1-91.4)	19.5% (16.6-22.8)	82.6% (81.4-83.8)
**65-69**	39.6% (33.4-45)	83.5% (82.4-84.5)	57.3% (54.1-60.8)	96.4% (95.7-97.1)	45.2% (39.3-50.7)	88.1% (86.8-89.3)	48% (41.5-54.5)	88.4% (87.1-89.7)	22.7% (19.4-26.4)	79.7% (78.3-81.1)
**70-74**	46.4% (39.9-51.9)	79.3% (78-80.5)	64% (60.9-67.2)	95.3% (94.4-96.2)	52.2% (46.1-57.7)	84.9% (83.3-86.3)	54.9% (48.4-61.3)	85.2% (83.6-86.9)	27.9% (24.2-32.1)	74.8% (73.2-76.4)
**75-79**	52.6% (45.9-58)	75% (73.4-76.3)	69.4% (66.6-72.4)	94.1% (93-95.2)	58.2% (52.2-63.5)	81.5% (79.6-83.1)	60.9% (54.5-66.9)	81.8% (80-83.8)	33.1% (28.9-37.7)	69.9% (68.1-71.7)
**80-84**	58.1% (51.5-63.4)	70.6% (68.8-72)	74% (71.4-76.7)	92.7% (91.4-94.1)	63.6% (57.8-68.6)	77.8% (75.7-79.7)	66.1% (60-71.7)	78.3% (76.1-80.5)	38.3% (33.8-43.1)	65% (63-66.9)
**85-89**	63.2% (56.8-68.1)	66% (64.1-67.6)	77.8% (75.5-80.2)	91.2% (89.6-92.8)	68.3% (62.9-72.9)	74% (71.6-76.1)	70.7% (65-75.8)	74.4% (72.1-77)	43.4% (38.6-48.3)	60% (58-62.1)
**90-95**	67.8% (61.7-72.4)	61.3% (59.3-63)	81.2% (79.1-83.3)	89.4% (87.5-91.3)	72.6% (67.5-76.7)	69.9% (67.3-72.2)	74.7% (69.4-79.3)	70.4% (67.8-73.2)	48.5% (43.6-53.4)	55.1% (52.9-57.2)

Supplemental Table 24: Positive and Negative Predictive values of plasma biomarkers for amyloid-β pathology in individuals with Corticobasal syndrome

	p-tau181		p-tau217		p-tau231		GFAP		NfL	
**Age**	PPV (95%CI)	NPV (95%CI)	PPV (95%CI)	NPV (95%CI)	PPV (95%CI)	NPV (95%CI)	PPV (95%CI)	NPV (95%CI)	PPV (95%CI)	NPV (95%CI)
**50-54**	Prevalence data unavailable	Prevalence data unavailable	Prevalence data unavailable	Prevalence data unavailable	Prevalence data unavailable	Prevalence data unavailable	Prevalence data unavailable	Prevalence data unavailable	Prevalence data unavailable	Prevalence data unavailable
**55-59**	70.3% (64.5-74.7)	58.4% (56.4-60.1)	82.9% (81-84.9)	88.2% (86.1-90.3)	74.9% (70.1-78.8)	67.3% (64.6-69.7)	76.9% (71.9-81.2)	67.8% (65.1-70.8)	51.5% (46.5-56.4)	52.1% (49.9-54.2)
**60-64**	67.8% (61.7-72.4)	61.3% (59.3-63)	81.2% (79.1-83.3)	89.4% (87.5-91.3)	72.6% (67.5-76.7)	69.9% (67.3-72.2)	74.7% (69.4-79.3)	70.4% (67.8-73.2)	48.5% (43.6-53.4)	55.1% (52.9-57.2)
**65-69**	61.2% (54.7-66.3)	67.8% (66-69.4)	76.4% (73.9-78.9)	91.8% (90.3-93.3)	66.5% (60.9-71.2)	75.6% (73.2-77.6)	68.9% (63-74.2)	76% (73.7-78.4)	41.4% (36.7-46.3)	62% (60-64)
**70-74**	58.1% (51.5-63.4)	70.6% (68.8-72)	74% (71.4-76.7)	92.7% (91.4-94.1)	63.6% (57.8-68.6)	77.8% (75.7-79.7)	66.1% (60-71.7)	78.3% (76.1-80.5)	38.3% (33.8-43.1)	65% (63-66.9)
**75-79**	52.6% (45.9-58)	75% (73.4-76.3)	69.4% (66.6-72.4)	94.1% (93-95.2)	58.2% (52.2-63.5)	81.5% (79.6-83.1)	60.9% (54.5-66.9)	81.8% (80-83.8)	33.1% (28.9-37.7)	69.9% (68.1-71.7)
**80-84**	46.4% (39.9-51.9)	79.3% (78-80.5)	64% (60.9-67.2)	95.3% (94.4-96.2)	52.2% (46.1-57.7)	84.9% (83.3-86.3)	54.9% (48.4-61.3)	85.2% (83.6-86.9)	27.9% (24.2-32.1)	74.8% (73.2-76.4)
**85-89**	42.4% (36.1-47.9)	81.9% (80.6-82.9)	60.2% (57-63.6)	96% (95.2-96.8)	48.1% (42.1-53.6)	86.9% (85.4-88.1)	50.9% (44.3-57.3)	87.1% (85.7-88.6)	24.8% (21.3-28.7)	77.8% (76.2-79.2)
**90-95**	38.1% (32.1-43.5)	84.4% (83.2-85.3)	55.8% (52.6-59.3)	96.6% (96-97.3)	43.7% (37.8-49.2)	88.8% (87.5-89.8)	46.5% (40-52.9)	89% (87.8-90.3)	21.6% (18.5-25.2)	80.7% (79.3-82)

Supplemental Table 25: Positive and Negative predictive values of plasma p-tau217 for amyloid-β pathology in different neurodegenerative syndromes

	MCI		Probable AD dementia		Frontotemporal dementia		Vascular dementia		Corticobasal syndrome	
**Age**	PPV % (95%CI)	NPV % (95%CI)	PPV % (95%CI)	NPV % (95%CI)	PPV % (95%CI)	NPV % (95%CI)	PPV % (95%CI)	NPV % (95%CI)	PPV % (95%CI)	NPV % (95%CI)
**50-54**	68.4% (65.5-71.4)	94.4% (93.3-95.4)	97.5% (96.5-98.1)	44.6% (40.1-50)	27.6% (25-30.5)	99% (98.8-99.2)	45.2% (41.9-48.7)	97.8% (97.3-98.2)	Prevalence data unavailable	Prevalence data unavailable
**55-59**	73.1% (70.5-75.9)	93% (91.7-94.3)	97.4% (96.3-98)	45.7% (41.2-51.1)	30.5% (27.8-33.7)	98.8% (98.6-99)	49.1% (45.8-52.7)	97.4% (96.9-97.9)	82.9% (81-84.9)	88.2% (86.1-90.3)
**60-64**	77.1% (74.7-79.6)	91.5% (89.9-93)	97.3% (96.1-97.9)	47% (42.4-52.4)	33.3% (30.5-36.6)	98.6% (98.4-98.9)	52.6% (49.3-56.2)	97% (96.4-97.6)	81.2% (79.1-83.3)	89.4% (87.5-91.3)
**65-69**	80.9% (78.7-83)	89.6% (87.7-91.4)	97% (95.7-97.7)	49.5% (44.8-54.8)	40.8% (37.7-44.3)	98.1% (97.8-98.5)	57.3% (54.1-60.8)	96.4% (95.7-97.1)	76.4% (73.9-78.9)	91.8% (90.3-93.3)
**70-74**	84% (82.2-85.9)	87.3% (85.1-89.5)	96.7% (95.4-97.5)	51.7% (47.1-57.1)	47.2% (43.9-50.7)	97.6% (97.1-98.1)	64% (60.9-67.2)	95.3% (94.4-96.2)	74% (71.4-76.7)	92.7% (91.4-94.1)
**75-79**	87.5% (86-89)	83.9% (81.2-86.6)	96.5% (95-97.3)	53.9% (49.3-59.2)	52.6% (49.3-56.2)	97% (96.4-97.6)	69.4% (66.6-72.4)	94.1% (93-95.2)	69.4% (66.6-72.4)	94.1% (93-95.2)
**80-84**	89.6% (88.3-90.9)	80.8% (77.8-83.9)	96.2% (94.6-97.1)	55.9% (51.3-61.1)	57.3% (54.1-60.8)	96.4% (95.7-97.1)	74% (71.4-76.7)	92.7% (91.4-94.1)	64% (60.9-67.2)	95.3% (94.4-96.2)
**85-89**	91.1% (90-92.2)	77.9% (74.6-81.4)	95.7% (93.9-96.7)	58.6% (54.1-63.7)	61.5% (58.3-64.8)	95.8% (95-96.6)	77.8% (75.5-80.2)	91.2% (89.6-92.8)	60.2% (57-63.6)	96% (95.2-96.8)
**90-95**	92.5% (91.6-93.5)	74.6% (70.9-78.4)	95.3% (93.3-96.4)	61.2% (56.7-66.1)	65.2% (62.1-68.3)	95.1% (94.2-96)	81.2% (79.1-83.3)	89.4% (87.5-91.3)	55.8% (52.6-59.3)	96.6% (96-97.3)

Supplemental Table 26: Positive and Negative Predictive values of plasma biomarkers for amyloid-β pathology in individuals with frontotemporal dementia who are APOEε4 noncarriers

	p-tau181		p-tau217		p-tau231		GFAP		NfL	
**Age**	PPV (95%CI)	NPV (95%CI)	PPV (95%CI)	NPV (95%CI)	PPV (95%CI)	NPV (95%CI)	PPV (95%CI)	NPV (95%CI)	PPV (95%CI)	NPV (95%CI)
**50-54**	Prevalence data unavailable	Prevalence data unavailable	Prevalence data unavailable	Prevalence data unavailable	Prevalence data unavailable	Prevalence data unavailable	Prevalence data unavailable	Prevalence data unavailable	Prevalence data unavailable	Prevalence data unavailable
**55-59**	11.5% (9-13.9)	96.2% (95.9-96.5)	21% (18.9-23.5)	99.3% (99.1-99.4)	14% (11.4-16.9)	97.4% (97.1-97.7)	15.4% (12.3-19.1)	97.5% (97.2-97.8)	5.5% (4.6-6.6)	95.2% (94.8-95.6)
**60-64**	13.6% (10.8-16.4)	95.5% (95.1-95.8)	24.4% (22.1-27.2)	99.1% (98.9-99.3)	16.5% (13.5-19.8)	96.9% (96.5-97.2)	18.1% (14.5-22.3)	96.9% (96.6-97.3)	6.6% (5.5-7.9)	94.2% (93.8-94.7)
**65-69**	17.7% (14.1-21.1)	93.9% (93.5-94.3)	30.5% (27.8-33.7)	98.8% (98.6-99)	21.3% (17.5-25.2)	95.8% (95.3-96.2)	23.2% (18.8-28.1)	95.9% (95.4-96.4)	8.8% (7.3-10.5)	92.3% (91.7-92.9)
**70-74**	19.6% (15.7-23.3)	93.2% (92.6-93.6)	33.3% (30.5-36.6)	98.6% (98.4-98.9)	23.5% (19.4-27.7)	95.2% (94.7-95.7)	25.6% (20.9-30.8)	95.3% (94.8-95.9)	9.8% (8.2-11.7)	91.3% (90.6-92)
**75-79**	25.2% (20.5-29.6)	90.8% (90.1-91.4)	40.8% (37.7-44.3)	98.1% (97.8-98.5)	29.7% (24.9-34.6)	93.5% (92.8-94.2)	32.1% (26.7-38)	93.7% (92.9-94.5)	13.1% (11-15.5)	88.4% (87.5-89.3)
**80-84**	31.2% (25.7-36.1)	88% (87.1-88.8)	48.1% (44.9-51.7)	97.5% (97-98)	36.3% (30.9-41.5)	91.5% (90.5-92.3)	38.9% (32.8-45.2)	91.7% (90.7-92.7)	16.8% (14.3-19.8)	85.1% (83.9-86.1)
**85-89**	35.1% (29.3-40.3)	86% (85-86.9)	52.6% (49.3-56.2)	97% (96.4-97.6)	40.5% (34.8-46)	90% (88.9-91)	43.2% (36.9-49.7)	90.2% (89.1-91.4)	19.5% (16.6-22.8)	82.6% (81.4-83.8)
**90-95**	Prevalence data unavailable	Prevalence data unavailable	Prevalence data unavailable	Prevalence data unavailable	Prevalence data unavailable	Prevalence data unavailable	Prevalence data unavailable	Prevalence data unavailable	Prevalence data unavailable	Prevalence data unavailable

Supplemental Table 27: Positive and Negative Predictive values of plasma biomarkers for amyloid-β pathology in individuals with Vascular dementia who are APOEε4 noncarriers

	p-tau181		p-tau217		p-tau231		GFAP		NfL	
**Age**	PPV (95%CI)	NPV (95%CI)	PPV (95%CI)	NPV (95%CI)	PPV (95%CI)	NPV (95%CI)	PPV (95%CI)	NPV (95%CI)	PPV (95%CI)	NPV (95%CI)
**50-54**	Prevalence data unavailable	Prevalence data unavailable	Prevalence data unavailable	Prevalence data unavailable	Prevalence data unavailable	Prevalence data unavailable	Prevalence data unavailable	Prevalence data unavailable	Prevalence data unavailable	Prevalence data unavailable
**55-59**	11.5% (9-13.9)	96.2% (95.9-96.5)	21% (18.9-23.5)	99.3% (99.1-99.4)	14% (11.4-16.9)	97.4% (97.1-97.7)	15.4% (12.3-19.1)	97.5% (97.2-97.8)	5.5% (4.6-6.6)	95.2% (94.8-95.6)
**60-64**	17.7% (14.1-21.1)	93.9% (93.5-94.3)	30.5% (27.8-33.7)	98.8% (98.6-99)	21.3% (17.5-25.2)	95.8% (95.3-96.2)	23.2% (18.8-28.1)	95.9% (95.4-96.4)	8.8% (7.3-10.5)	92.3% (91.7-92.9)
**65-69**	21.5% (17.3-25.5)	92.4% (91.8-92.9)	36% (33-39.3)	98.5% (98.2-98.8)	25.7% (21.3-30.1)	94.7% (94-95.2)	27.8% (22.9-33.3)	94.8% (94.2-95.4)	10.9% (9.1-13)	90.4% (89.6-91.1)
**70-74**	32% (26.5-37)	87.6% (86.7-88.4)	49.1% (45.8-52.7)	97.4% (96.9-97.9)	37.2% (31.7-42.5)	91.2% (90.2-92.1)	39.8% (33.7-46.2)	91.4% (90.4-92.4)	17.4% (14.7-20.4)	84.6% (83.4-85.7)
**75-79**	39.6% (33.4-45)	83.5% (82.4-84.5)	57.3% (54.1-60.8)	96.4% (95.7-97.1)	45.2% (39.3-50.7)	88.1% (86.8-89.3)	48% (41.5-54.5)	88.4% (87.1-89.7)	22.7% (19.4-26.4)	79.7% (78.3-81.1)
**80-84**	46.4% (39.9-51.9)	79.3% (78-80.5)	64% (60.9-67.2)	95.3% (94.4-96.2)	52.2% (46.1-57.7)	84.9% (83.3-86.3)	54.9% (48.4-61.3)	85.2% (83.6-86.9)	27.9% (24.2-32.1)	74.8% (73.2-76.4)
**85-89**	61.2% (54.7-66.3)	67.8% (66-69.4)	76.4% (73.9-78.9)	91.8% (90.3-93.3)	66.5% (60.9-71.2)	75.6% (73.2-77.6)	68.9% (63-74.2)	76% (73.7-78.4)	41.4% (36.7-46.3)	62% (60-64)
**90-95**	69.5% (63.6-74)	59.4% (57.4-61.1)	82.4% (80.4-84.3)	88.6% (86.6-90.6)	74.1% (69.2-78.2)	68.2% (65.5-70.5)	76.2% (71.1-80.6)	68.7% (66-71.6)	50.5% (45.5-55.4)	53.1% (50.9-55.2)

Supplemental Table 28: Positive and Negative Predictive values of plasma biomarkers for amyloid-β pathology in individuals with Corticobasal syndrome who are APOEε4 noncarriers

	p-tau181		p-tau217		p-tau231		GFAP		NfL	
**Age**	PPV (95%CI)	NPV (95%CI)	PPV (95%CI)	NPV (95%CI)	PPV (95%CI)	NPV (95%CI)	PPV (95%CI)	NPV (95%CI)	PPV (95%CI)	NPV (95%CI)
**50-54**	Prevalence data unavailable	Prevalence data unavailable	Prevalence data unavailable	Prevalence data unavailable	Prevalence data unavailable	Prevalence data unavailable	Prevalence data unavailable	Prevalence data unavailable	Prevalence data unavailable	Prevalence data unavailable
**55-59**	58.1% (51.5-63.4)	70.6% (68.8-72)	74% (71.4-76.7)	92.7% (91.4-94.1)	63.6% (57.8-68.6)	77.8% (75.7-79.7)	66.1% (60-71.7)	78.3% (76.1-80.5)	38.3% (33.8-43.1)	65% (63-66.9)
**60-64**	54.9% (48.2-60.2)	73.3% (71.6-74.6)	71.4% (68.6-74.2)	93.6% (92.4-94.8)	60.5% (54.5-65.6)	80% (78-81.8)	63.1% (56.8-68.9)	80.4% (78.5-82.5)	35.2% (30.9-39.9)	67.9% (66-69.8)
**65-69**	51.4% (44.7-56.9)	75.9% (74.4-77.2)	68.4% (65.5-71.4)	94.4% (93.3-95.4)	57.1% (51.1-62.4)	82.2% (80.3-83.8)	59.8% (53.3-65.9)	82.5% (80.7-84.4)	32.1% (28-36.6)	70.9% (69.1-72.7)
**70-74**	47.7% (41.1-53.2)	78.5% (77.1-79.7)	65.2% (62.1-68.3)	95.1% (94.2-96)	53.5% (47.4-58.9)	84.2% (82.6-85.7)	56.2% (49.7-62.5)	84.6% (82.9-86.3)	29% (25.1-33.2)	73.8% (72.1-75.5)
**75-79**	45.1% (38.6-50.6)	80.2% (78.8-81.3)	62.8% (59.7-66.1)	95.6% (94.7-96.4)	50.9% (44.8-56.4)	85.6% (84-86.9)	53.6% (47.1-60)	85.9% (84.3-87.5)	26.9% (23.2-31)	75.8% (74.2-77.4)
**80-84**	42.4% (36.1-47.9)	81.9% (80.6-82.9)	60.2% (57-63.6)	96% (95.2-96.8)	48.1% (42.1-53.6)	86.9% (85.4-88.1)	50.9% (44.3-57.3)	87.1% (85.7-88.6)	24.8% (21.3-28.7)	77.8% (76.2-79.2)
**85-89**	39.6% (33.4-45)	83.5% (82.4-84.5)	57.3% (54.1-60.8)	96.4% (95.7-97.1)	45.2% (39.3-50.7)	88.1% (86.8-89.3)	48% (41.5-54.5)	88.4% (87.1-89.7)	22.7% (19.4-26.4)	79.7% (78.3-81.1)
**90-95**	36.7% (30.7-41.9)	85.2% (84.1-86.1)	54.3% (51-57.8)	96.8% (96.2-97.4)	42.1% (36.4-47.6)	89.4% (88.2-90.4)	44.9% (38.5-51.4)	89.6% (88.4-90.8)	20.6% (17.5-24)	81.7% (80.3-82.9)

Supplemental Table 29: Positive and Negative Predictive values of plasma biomarkers for amyloid-β pathology in individuals with frontotemporal dementia who are APOEε4 carriers

	p-tau181		p-tau217		p-tau231		GFAP		NfL	
**Age**	PPV (95%CI)	NPV (95%CI)	PPV (95%CI)	NPV (95%CI)	PPV (95%CI)	NPV (95%CI)	PPV (95%CI)	NPV (95%CI)	PPV (95%CI)	NPV (95%CI)
**50-54**	23.4% (18.9-27.6)	91.6% (91-92.1)	38.5% (35.4-41.9)	98.3% (98-98.6)	27.7% (23.1-32.4)	94.1% (93.4-94.7)	30% (24.8-35.7)	94.2% (93.6-95)	12% (10.1-14.3)	89.4% (88.6-90.2)
**55-59**	29.5% (24.3-34.3)	88.8% (88-89.5)	46.2% (42.9-49.8)	97.7% (97.2-98.1)	34.5% (29.2-39.7)	92.1% (91.2-92.9)	37.1% (31.1-43.3)	92.3% (91.4-93.2)	15.8% (13.3-18.6)	86% (85-87)
**60-64**	36.7% (30.7-41.9)	85.2% (84.1-86.1)	54.3% (51-57.8)	96.8% (96.2-97.4)	42.1% (36.4-47.6)	89.4% (88.2-90.4)	44.9% (38.5-51.4)	89.6% (88.4-90.8)	20.6% (17.5-24)	81.7% (80.3-82.9)
**65-69**	42.4% (36.1-47.9)	81.9% (80.6-82.9)	60.2% (57-63.6)	96% (95.2-96.8)	48.1% (42.1-53.6)	86.9% (85.4-88.1)	50.9% (44.3-57.3)	87.1% (85.7-88.6)	24.8% (21.3-28.7)	77.8% (76.2-79.2)
**70-74**	49% (42.3-54.5)	77.6% (76.2-78.9)	66.3% (63.3-69.4)	94.9% (93.9-95.8)	54.7% (48.6-60.1)	83.6% (81.8-85)	57.4% (50.9-63.6)	83.9% (82.2-85.7)	30% (26.1-34.4)	72.9% (71.1-74.5)
**75-79**	58.1% (51.5-63.4)	70.6% (68.8-72)	74% (71.4-76.7)	92.7% (91.4-94.1)	63.6% (57.8-68.6)	77.8% (75.7-79.7)	66.1% (60-71.7)	78.3% (76.1-80.5)	38.3% (33.8-43.1)	65% (63-66.9)
**80-84**	64.1% (57.8-69)	65.1% (63.2-66.7)	78.6% (76.3-80.9)	90.8% (89.2-92.5)	69.2% (63.8-73.7)	73.2% (70.7-75.3)	71.5% (65.9-76.5)	73.7% (71.3-76.2)	44.4% (39.6-49.4)	59% (56.9-61.1)
**85-89**	Prevalence data unavailable	Prevalence data unavailable	Prevalence data unavailable	Prevalence data unavailable	Prevalence data unavailable	Prevalence data unavailable	Prevalence data unavailable	Prevalence data unavailable	Prevalence data unavailable	Prevalence data unavailable
**90-95**	Prevalence data unavailable	Prevalence data unavailable	Prevalence data unavailable	Prevalence data unavailable	Prevalence data unavailable	Prevalence data unavailable	Prevalence data unavailable	Prevalence data unavailable	Prevalence data unavailable	Prevalence data unavailable

Supplemental Table 30: Positive and Negative Predictive values of plasma biomarkers for amyloid-β pathology in individuals with Vascular dementia who are APOEε4 carriers

	p-tau181		p-tau217		p-tau231		GFAP		NfL	
**Age**	PPV (95%CI)	NPV (95%CI)	PPV (95%CI)	NPV (95%CI)	PPV (95%CI)	NPV (95%CI)	PPV (95%CI)	NPV (95%CI)	PPV (95%CI)	NPV (95%CI)
**50-54**	Prevalence data unavailable	Prevalence data unavailable	Prevalence data unavailable	Prevalence data unavailable	Prevalence data unavailable	Prevalence data unavailable	Prevalence data unavailable	Prevalence data unavailable	Prevalence data unavailable	Prevalence data unavailable
**55-59**	35.1% (29.3-40.3)	86% (85-86.9)	52.6% (49.3-56.2)	97% (96.4-97.6)	40.5% (34.8-46)	90% (88.9-91)	43.2% (36.9-49.7)	90.2% (89.1-91.4)	19.5% (16.6-22.8)	82.6% (81.4-83.8)
**60-64**	46.4% (39.9-51.9)	79.3% (78-80.5)	64% (60.9-67.2)	95.3% (94.4-96.2)	52.2% (46.1-57.7)	84.9% (83.3-86.3)	54.9% (48.4-61.3)	85.2% (83.6-86.9)	27.9% (24.2-32.1)	74.8% (73.2-76.4)
**65-69**	57% (50.4-62.4)	71.5% (69.8-72.9)	73.1% (70.5-75.9)	93% (91.7-94.3)	62.6% (56.7-67.6)	78.6% (76.5-80.4)	65.1% (58.9-70.8)	79% (76.9-81.2)	37.3% (32.8-42)	66% (64-67.9)
**70-74**	66% (59.7-70.7)	63.2% (61.3-64.9)	79.9% (77.7-82.1)	90.1% (88.4-91.9)	70.9% (65.7-75.3)	71.5% (69-73.8)	73.2% (67.7-78)	72% (69.6-74.7)	46.4% (41.6-51.4)	57.1% (54.9-59.2)
**75-79**	76.6% (71.5-80.3)	50.4% (48.4-52.2)	87% (85.5-88.6)	84.4% (81.8-87)	80.5% (76.3-83.7)	59.8% (56.9-62.5)	82.1% (77.9-85.6)	60.4% (57.5-63.7)	59.4% (54.6-64.1)	44.1% (41.9-46.2)
**80-84**	81.4% (77.1-84.5)	43.1% (41.1-44.9)	90% (88.7-91.2)	80.1% (77-83.3)	84.7% (81.2-87.3)	52.6% (49.6-55.4)	86.1% (82.6-88.9)	53.2% (50.2-56.7)	66.2% (61.7-70.5)	37% (35-39)
**85-89**	87.5% (84.3-89.8)	32.2% (30.4-33.8)	93.5% (92.7-94.3)	71.6% (67.7-75.8)	89.8% (87.4-91.7)	41% (38.1-43.7)	90.8% (88.4-92.8)	41.6% (38.7-45)	75.8% (72.1-79.3)	26.8% (25.2-28.6)
**90-95**	Prevalence data unavailable	Prevalence data unavailable	Prevalence data unavailable	Prevalence data unavailable	Prevalence data unavailable	Prevalence data unavailable	Prevalence data unavailable	Prevalence data unavailable	Prevalence data unavailable	Prevalence data unavailable

Supplemental Table 31: Positive and Negative Predictive values of plasma biomarkers for amyloid-β pathology in individuals with Corticobasal syndrome who are APOEε4 carriers

	p-tau181		p-tau217		p-tau231		GFAP		NfL	
**Age**	PPV (95%CI)	NPV (95%CI)	PPV (95%CI)	NPV (95%CI)	PPV (95%CI)	NPV (95%CI)	PPV (95%CI)	NPV (95%CI)	PPV (95%CI)	NPV (95%CI)
**50-54**	51.3% (84.3-89.8)	33% (30.4-33.8)	93.5% (92.7-94.3)	71.6% (67.7-75.8)	89.8% (87.4-91.7)	41% (38.1-43.7)	90.8% (88.4-92.8)	41.6% (38.7-45)	75.8% (72.1-79.3)	26.8% (25.2-28.6)
**55-59**	85.8% (82.2-88.3)	35.5% (33.7-37.2)	92.5% (91.6-93.5)	74.6% (70.9-78.4)	88.4% (85.6-90.5)	44.7% (41.7-47.4)	89.5% (86.7-91.7)	45.3% (42.3-48.7)	73% (68.9-76.7)	29.9% (28.1-31.7)
**60-64**	84% (80.1-86.7)	38.8% (36.9-40.6)	91.5% (90.4-92.5)	77.1% (73.7-80.7)	86.8% (83.8-89.2)	48.2% (45.2-51)	88.1% (85-90.5)	48.8% (45.8-52.2)	70.1% (65.8-74.1)	32.9% (31.1-34.9)
**65-69**	82.7% (78.6-85.7)	41% (39-42.8)	90.8% (89.6-91.9)	78.7% (75.4-82.1)	85.8% (82.5-88.3)	50.4% (47.4-53.2)	87.1% (83.8-89.7)	51% (48.1-54.5)	68.2% (63.8-72.3)	35% (33-37)
**70-74**	80.8% (76.3-84)	44.2% (42.2-46)	89.6% (88.3-90.9)	80.8% (77.8-83.9)	84.1% (80.6-86.8)	53.7% (50.7-56.5)	85.5% (82-88.5)	54.3% (51.3-57.7)	65.3% (60.7-69.6)	38% (36-40.1)
**75-79**	79.1% (74.3-82.5)	46.8% (44.8-48.6)	88.6% (87.2-89.9)	82.4% (79.6-85.3)	82.6% (78.9-85.6)	56.3% (53.3-59.1)	84.2% (80.3-87.3)	56.9% (54-60.3)	62.8% (58.1-67.3)	40.5% (38.5-42.6)
**80-84**	77.3% (72.3-80.9)	49.4% (47.4-51.2)	87.5% (86-89)	83.9% (81.2-86.6)	81.1% (77.1-84.3)	58.9% (55.9-61.5)	82.7% (78.6-86.1)	59.4% (56.5-62.7)	60.4% (55.6-65)	43.1% (41-45.2)
**85-89**	Prevalence data unavailable	Prevalence data unavailable	Prevalence data unavailable	Prevalence data unavailable	Prevalence data unavailable	Prevalence data unavailable	Prevalence data unavailable	Prevalence data unavailable	Prevalence data unavailable	Prevalence data unavailable
**90-95**	Prevalence data unavailable	Prevalence data unavailable	Prevalence data unavailable	Prevalence data unavailable	Prevalence data unavailable	Prevalence data unavailable	Prevalence data unavailable	Prevalence data unavailable	Prevalence data unavailable	Prevalence data unavailable

Supplemental Table 32: Positive and Negative predictive values of plasma p-tau217 for amyloid-β pathology in different clinical syndromes APOE4 carriers.

	Mild Cognitive Impairment		Probable AD dementia		Frontotemporal dementia		Vascular dementia		Corticobasal syndrome	
**Age**	PPV % (95%CI)	NPV % (95%CI)	PPV % (95%CI)	NPV % (95%CI)	PPV % (95%CI)	NPV % (95%CI)	PPV % (95%CI)	NPV % (95%CI)	PPV % (95%CI)	NPV % (95%CI)
**50-54**	69.4% (66.6-72.4)	94.1% (93-95.2)	96.6% (96.2-97.1)	55.9% (51.3-61.1)	38.5% (35.4-41.9)	98.3% (98-98.6)	Prevalence data unavailable	Prevalence data unavailable	93.5% (92.7-94.3)	71.6% (67.7-75.8)
**55-59**	75.6% (73.1-78.2)	92.1% (90.7-93.6)	96.7% (96.2-97.1)	55.4% (50.8-60.6)	46.2% (42.9-49.8)	97.7% (97.2-98.1)	52.6% (49.3-56.2)	97% (96.4-97.6)	92.5% (91.6-93.5)	74.6% (70.9-78.4)
**60-64**	82.4% (80.4-84.3)	88.6% (86.6-90.6)	96.8% (96.3-97.2)	54.9% (50.3-60.1)	54.3% (51-57.8)	96.8% (96.2-97.4)	64% (60.9-67.2)	95.3% (94.4-96.2)	91.5% (90.4-92.5)	77.1% (73.7-80.7)
**65-69**	86.6% (85-88.1)	84.9% (82.4-87.5)	96.8% (96.4-97.2)	54.4% (49.8-59.6)	60.2% (57-63.6)	96% (95.2-96.8)	73.1% (70.5-75.9)	93% (91.7-94.3)	90.8% (89.6-91.9)	78.7% (75.4-82.1)
**70-74**	90.8% (89.6-91.9)	78.7% (75.4-82.1)	96.9% (96.5-97.3)	53.9% (49.3-59.2)	66.3% (63.3-69.4)	94.9% (93.9-95.8)	79.9% (77.7-82.1)	90.1% (88.4-91.9)	89.6% (88.3-90.9)	80.8% (77.8-83.9)
**75-79**	93.5% (92.7-94.3)	71.6% (67.7-75.8)	97.1% (96.7-97.5)	51.7% (47.1-57.1)	74% (71.4-76.7)	92.7% (91.4-94.1)	87% (85.5-88.6)	84.4% (81.8-87)	88.6% (87.2-89.9)	82.4% (79.6-85.3)
**80-84**	95.6% (95-96.1)	62.7% (58.3-67.6)	97.4% (97-97.7)	49.5% (44.8-54.8)	78.6% (76.3-80.9)	90.8% (89.2-92.5)	90% (88.7-91.2)	80.1% (77-83.3)	87.5% (86-89)	83.9% (81.2-86.6)
**85-89**	97.1% (96.7-97.5)	51.7% (47.1-57.1)	97.5% (97.2-97.8)	48.2% (43.7-53.6)	Prevalence data unavailable	Prevalence data unavailable	93.5% (92.7-94.3)	71.6% (67.7-75.8)	Prevalence data unavailable	Prevalence data unavailable
**90-95**	97.9% (97.6-98.1)	44.4% (39.9-49.7)	97.6% (97.3-97.9)	47% (42.4-52.4)	Prevalence data unavailable	Prevalence data unavailable	Prevalence data unavailable	Prevalence data unavailable	Prevalence data unavailable	Prevalence data unavailable

Supplemental Table 33: Positive and Negative predictive values of plasma p-tau217 for amyloid-β pathology in different clinical syndromes who are APOE4 noncarriers.

	Mild Cognitive Impairment		Probable AD dementia		Frontotemporal dementia		Vascular dementia		Corticobasal syndrome	
**Age**	PPV % (95%CI)	NPV % (95%CI)	PPV % (95%CI)	NPV % (95%CI)	PPV % (95%CI)	NPV % (95%CI)	PPV % (95%CI)	NPV % (95%CI)	PPV % (95%CI)	NPV % (95%CI)
**50-54**	50.9% (47.6-54.5)	97.2% (96.7-97.7)	94.7% (94-95.4)	66.9% (62.7-71.5)	Prevalence data unavailable	Prevalence data unavailable	Prevalence data unavailable	Prevalence data unavailable	Prevalence data unavailable	Prevalence data unavailable
**55-59**	55.8% (52.6-59.3)	96.6% (96-97.3)	94.9% (94.2-95.5)	66.3% (62-70.9)	21% (18.9-23.5)	99.3% (99.1-99.4)	21% (18.9-23.5)	99.3% (99.1-99.4)	74% (71.4-76.7)	92.7% (91.4-94.1)
**60-64**	60.2% (57-63.6)	96% (95.2-96.8)	95.1% (94.5-95.8)	64.9% (60.6-69.6)	24.4% (22.1-27.2)	99.1% (98.9-99.3)	30.5% (27.8-33.7)	98.8% (98.6-99)	71.4% (68.6-74.2)	93.6% (92.4-94.8)
**65-69**	65.2% (62.1-68.3)	95.1% (94.2-96)	95.2% (94.6-95.8)	64.6% (60.2-69.3)	30.5% (27.8-33.7)	98.8% (98.6-99)	36% (33-39.3)	98.5% (98.2-98.8)	68.4% (65.5-71.4)	94.4% (93.3-95.4)
**70-74**	71.4% (68.6-74.2)	93.6% (92.4-94.8)	95.3% (94.7-95.9)	64.2% (59.9-69)	33.3% (30.5-36.6)	98.6% (98.4-98.9)	49.1% (45.8-52.7)	97.4% (96.9-97.9)	65.2% (62.1-68.3)	95.1% (94.2-96)
**75-79**	76.4% (73.9-78.9)	91.8% (90.3-93.3)	95.4% (94.7-96)	63.8% (59.5-68.6)	40.8% (37.7-44.3)	98.1% (97.8-98.5)	57.3% (54.1-60.8)	96.4% (95.7-97.1)	62.8% (59.7-66.1)	95.6% (94.7-96.4)
**80-84**	80.5% (78.4-82.7)	89.8% (87.9-91.6)	95.4% (94.8-96)	63.5% (59.1-68.3)	48.1% (44.9-51.7)	97.5% (97-98)	64% (60.9-67.2)	95.3% (94.4-96.2)	60.2% (57-63.6)	96% (95.2-96.8)
**85-89**	84% (82.2-85.9)	87.3% (85.1-89.5)	95.6% (95-96.1)	62.7% (58.3-67.6)	52.6% (49.3-56.2)	97% (96.4-97.6)	76.4% (73.9-78.9)	91.8% (90.3-93.3)	57.3% (54.1-60.8)	96.4% (95.7-97.1)
**90-95**	87% (85.5-88.6)	84.4% (81.8-87)	95.8% (95.3-96.4)	61.2% (56.7-66.1)	Prevalence data unavailable	Prevalence data unavailable	82.4% (80.4-84.3)	88.6% (86.6-90.6)	54.3% (51-57.8)	96.8% (96.2-97.4)

Supplemental Table 34: Positive and Negative Predictive values of plasma biomarkers for amyloid-β pathology in individuals with MCI using the lower estimate of amyloid-β pathology prevalence.

	p-tau181		p-tau217		p-tau231		GFAP		NfL	
**Age**	PPV (95%CI)	NPV (95%CI)	PPV (95%CI)	NPV (95%CI)	PPV (95%CI)	NPV (95%CI)	PPV (95%CI)	NPV (95%CI)	PPV (95%CI)	NPV (95%CI)
**50-54**	41.9% (38.9-44.9)	82.2% (79.2-85.2)	59.6% (56.6-62.6)	96.1% (93.1-99.1)	47.6% (44.6-50.6)	87.1% (84.1-90.1)	50.3% (47.3-53.3)	87.4% (84.4-90.4)	24.4% (21.4-27.4)	78.1% (75.1-81.1)
**55-59**	48.5% (45.5-51.5)	78% (75-81)	65.8% (62.8-68.8)	95% (92-98)	54.2% (51.2-57.2)	83.8% (80.8-86.8)	57% (54-60)	84.2% (81.2-87.2)	29.6% (26.6-32.6)	73.3% (70.3-76.3)
**60-64**	55.4% (52.4-58.4)	72.8% (69.8-75.8)	71.8% (68.8-74.8)	93.4% (90.4-96.4)	61% (58-64)	79.7% (76.7-82.7)	63.6% (60.6-66.6)	80.1% (77.1-83.1)	35.7% (32.7-38.7)	67.5% (64.5-70.5)
**65-69**	62% (59-65)	67.1% (64.1-70.1)	77% (74-80)	91.6% (88.6-94.6)	67.2% (64.2-70.2)	74.9% (71.9-77.9)	69.6% (66.6-72.6)	75.4% (72.4-78.4)	42.2% (39.2-45.2)	61.2% (58.2-64.2)
**70-74**	67.8% (64.8-70.8)	61.2% (58.2-64.2)	81.2% (78.2-84.2)	89.3% (86.3-92.3)	72.6% (69.6-75.6)	69.8% (66.8-72.8)	74.8% (71.8-77.8)	70.3% (67.3-73.3)	48.6% (45.6-51.6)	55% (52-58)
**75-79**	72.8% (69.8-75.8)	55.4% (52.4-58.4)	84.6% (81.6-87.6)	86.8% (83.8-89.8)	77.2% (74.2-80.2)	64.5% (61.5-67.5)	79.1% (76.1-82.1)	65% (62-68)	54.5% (51.5-57.5)	49% (46-52)
**80-84**	76.9% (73.9-79.9)	49.9% (46.9-52.9)	87.2% (84.2-90.2)	84.1% (81.1-87.1)	80.8% (77.8-83.8)	59.4% (56.4-62.4)	82.4% (79.4-85.4)	59.9% (56.9-62.9)	59.9% (56.9-62.9)	43.6% (40.6-46.6)
**85-89**	80.2% (77.2-83.2)	45.1% (42.1-48.1)	89.3% (86.3-92.3)	81.3% (78.3-84.3)	83.6% (80.6-86.6)	54.6% (51.6-57.6)	85.1% (82.1-88.1)	55.2% (52.2-58.2)	64.5% (61.5-67.5)	38.8% (35.8-41.8)
**90-95**	82.9% (79.9-85.9)	40.7% (37.7-43.7)	90.9% (87.9-93.9)	78.5% (75.5-81.5)	85.9% (82.9-88.9)	50.1% (47.1-53.1)	87.2% (84.2-90.2)	50.7% (47.7-53.7)	68.5% (65.5-71.5)	34.7% (31.7-37.7)

Supplemental Table 35: Positive and Negative Predictive values of plasma biomarkers for amyloid-β pathology in individuals with probable AD dementia using the lower estimate of amyloid-β pathology prevalence.

	**p-tau181**		**p-tau217**		**p-tau231**		**GFAP**		**NfL**	
**Age**	PPV (95%CI)	NPV (95%CI)	PPV (95%CI)	NPV (95%CI)	PPV (95%CI)	NPV (95%CI)	PPV (95%CI)	NPV (95%CI)	PPV (95%CI)	NPV (95%CI)
**50-54**	93.9% (90.9-96.9)	17.8% (14.8-20.8)	98.7% (95.7-99.8)	32.5% (29.5-35.5)	96.5% (93.5-97.6)	18.2% (15.2-21.2)	96.9% (93.9-98)	18.5% (15.5-21.5)	90.8% (87.8-91.9)	10.5% (7.5-13.5)
**55-59**	93.8% (90.8-96.8)	18.1% (15.1-21.1)	98.5% (95.5-99.7)	36.1% (33.1-39.1)	96.4% (93.4-97.6)	18.8% (15.8-21.8)	96.7% (93.7-97.9)	19.2% (16.2-22.2)	90.4% (87.4-91.6)	10.9% (7.9-13.9)
**60-64**	93.5% (90.5-96.5)	18.7% (15.7-21.7)	98.2% (95.2-99.7)	39.4% (36.4-42.4)	96.2% (93.2-97.7)	19.6% (16.6-22.6)	96.6% (93.6-98.1)	20% (17-23)	89.9% (86.9-91.4)	11.4% (8.4-14.4)
**65-69**	93.2% (90.2-96.2)	19.5% (16.5-22.5)	98% (95-99.5)	42.4% (39.4-45.4)	95.8% (92.8-97.3)	21.2% (18.2-24.2)	96.2% (93.2-97.7)	21.6% (18.6-24.6)	89% (86-90.5)	12.5% (9.5-15.5)
**70-74**	92.7% (89.7-95.7)	20.7% (17.7-23.7)	97.8% (94.8-99.3)	44.9% (41.9-47.9)	95.4% (92.4-96.9)	22.8% (19.8-25.8)	95.9% (92.9-97.4)	23.2% (20.2-26.2)	88.1% (85.1-89.6)	13.5% (10.5-16.5)
**75-79**	92.1% (89.1-95.1)	22.3% (19.3-25.3)	97.6% (94.6-99.1)	47% (44-50)	95% (92-96.5)	24.3% (21.3-27.3)	95.5% (92.5-97)	24.8% (21.8-27.8)	87.1% (84.1-88.6)	14.5% (11.5-17.5)
**80-84**	91.2% (88.2-94.2)	24.4% (21.4-27.4)	97.5% (94.5-99)	48.5% (45.5-51.5)	94.6% (91.6-96.1)	25.9% (22.9-28.9)	95.2% (92.2-96.7)	26.3% (23.3-29.3)	86.2% (83.2-87.7)	15.6% (12.6-18.6)
**85-89**	90% (87-93)	27% (24-30)	97.4% (94.4-98.9)	49.5% (46.5-52.5)	94% (91-95.5)	28.1% (25.1-31.1)	94.6% (91.6-96.1)	28.6% (25.6-31.6)	84.8% (81.8-86.3)	17.1% (14.1-20.1)
**90-95**	88.6% (85.6-91.6)	30% (27-33)	97.3% (94.3-98.8)	50.2% (47.2-53.2)	93.4% (90.4-94.9)	30.2% (27.2-33.2)	94.1% (91.1-95.6)	30.8% (27.8-33.8)	83.4% (80.4-84.9)	18.6% (15.6-21.6)

Supplemental Table 36: Positive and Negative Predictive values of plasma biomarkers for amyloid-β pathology in individuals with frontotemporal dementia using the lower estimate of amyloid-β pathology prevalence.

	p-tau181		p-tau217		p-tau231		GFAP		NfL	
**Age**	PPV (95%CI)	NPV (95%CI)	PPV (95%CI)	NPV (95%CI)	PPV (95%CI)	NPV (95%CI)	PPV (95%CI)	NPV (95%CI)	PPV (95%CI)	NPV (95%CI)
**50-54**	9.3% (6.3-12.3)	97% (94-100)	17.4% (14.4-20.4)	99.4% (96.4-99.6)	11.5% (8.5-14.5)	97.9% (94.9-99.4)	12.6% (9.6-15.6)	98% (95-99.5)	4.4% (1.4-7.4)	96.2% (93.2-99.2)
**55-59**	11.5% (8.5-14.5)	96.2% (93.2-99.2)	21% (18-24)	99.3% (96.3-99.6)	14% (11-17)	97.4% (94.4-99.4)	15.4% (12.4-18.4)	97.5% (94.5-99.4)	5.5% (2.5-8.5)	95.2% (92.2-98.2)
**60-64**	15.7% (12.7-18.7)	94.7% (91.7-97.7)	27.6% (24.6-30.6)	99% (96-99.6)	18.9% (15.9-21.9)	96.3% (93.3-99.3)	20.7% (17.7-23.7)	96.4% (93.4-99.4)	7.7% (4.7-10.7)	93.3% (90.3-96.3)
**65-69**	17.7% (14.7-20.7)	93.9% (90.9-96.9)	30.5% (27.5-33.5)	98.8% (95.8-99.6)	21.3% (18.3-24.3)	95.8% (92.8-98.8)	23.2% (20.2-26.2)	95.9% (92.9-98.9)	8.8% (5.8-11.8)	92.3% (89.3-95.3)
**70-74**	21.5% (18.5-24.5)	92.4% (89.4-95.4)	36% (33-39)	98.5% (95.5-99.6)	25.7% (22.7-28.7)	94.7% (91.7-97.7)	27.8% (24.8-30.8)	94.8% (91.8-97.8)	10.9% (7.9-13.9)	90.4% (87.4-93.4)
**75-79**	23.4% (20.4-26.4)	91.6% (88.6-94.6)	38.5% (35.5-41.5)	98.3% (95.3-99.6)	27.7% (24.7-30.7)	94.1% (91.1-97.1)	30% (27-33)	94.2% (91.2-97.2)	12% (9-15)	89.4% (86.4-92.4)
**80-84**	23.4% (20.4-26.4)	91.6% (88.6-94.6)	38.5% (35.5-41.5)	98.3% (95.3-99.6)	27.7% (24.7-30.7)	94.1% (91.1-97.1)	30% (27-33)	94.2% (91.2-97.2)	12% (9-15)	89.4% (86.4-92.4)
**85-89**	23.4% (20.4-26.4)	91.6% (88.6-94.6)	38.5% (35.5-41.5)	98.3% (95.3-99.6)	27.7% (24.7-30.7)	94.1% (91.1-97.1)	30% (27-33)	94.2% (91.2-97.2)	12% (9-15)	89.4% (86.4-92.4)
**90-95**	Prevalence data unavailable	Prevalence data unavailable	Prevalence data unavailable	Prevalence data unavailable	Prevalence data unavailable	Prevalence data unavailable	Prevalence data unavailable	Prevalence data unavailable	Prevalence data unavailable	Prevalence data unavailable

Supplemental Table 37: Positive and Negative Predictive values of plasma biomarkers for amyloid-β pathology in individuals with Vascular dementia using the lower estimate of amyloid-β pathology prevalence.

	p-tau181		p-tau217		p-tau231		GFAP		NfL	
**Age**	PPV (95%CI)	NPV (95%CI)	PPV (95%CI)	NPV (95%CI)	PPV (95%CI)	NPV (95%CI)	PPV (95%CI)	NPV (95%CI)	PPV (95%CI)	NPV (95%CI)
**50-54**	9.3% (6.3-12.3)	97% (94-99.2)	17.4% (14.4-20.4)	99.4% (96.4-99.9)	11.5% (8.5-14.5)	97.9% (94.9-99.2)	12.6% (9.6-15.6)	98% (95-101)	4.4% (1.4-7.4)	96.2% (93.2-99.2)
**55-59**	13.6% (10.6-16.6)	95.5% (92.5-98.5)	24.4% (21.4-27.4)	99.1% (96.1-99.8)	16.5% (13.5-19.5)	96.9% (93.9-99.2)	18.1% (15.1-21.1)	96.9% (93.9-99.9)	6.6% (3.6-9.6)	94.2% (91.2-97.2)
**60-64**	19.6% (16.6-22.6)	93.2% (90.2-96.2)	33.3% (30.3-36.3)	98.6% (95.6-99.6)	23.5% (20.5-26.5)	95.2% (92.2-98.2)	25.6% (22.6-28.6)	95.3% (92.3-98.3)	9.8% (6.8-12.8)	91.3% (88.3-94.3)
**65-69**	28.7% (25.7-31.7)	89.2% (86.2-92.2)	45.2% (42.2-48.2)	97.8% (94.8-98.8)	33.6% (30.6-36.6)	92.4% (89.4-95.4)	36.1% (33.1-39.1)	92.6% (89.6-95.6)	15.2% (12.2-18.2)	86.5% (83.5-89.5)
**70-74**	35.1% (32.1-38.1)	86% (83-89)	52.6% (49.6-55.6)	97% (94-98.4)	40.5% (37.5-43.5)	90% (87-93)	43.2% (40.2-46.2)	90.2% (87.2-93.2)	19.5% (16.5-22.5)	82.6% (79.6-85.6)
**75-79**	42.4% (39.4-45.4)	81.9% (78.9-84.9)	60.2% (57.2-63.2)	96% (93-98.1)	48.1% (45.1-51.1)	86.9% (83.9-89.9)	50.9% (47.9-53.9)	87.1% (84.1-90.1)	24.8% (21.8-27.8)	77.8% (74.8-80.8)
**80-84**	47.7% (44.7-50.7)	78.5% (75.5-81.5)	65.2% (62.2-68.2)	95.1% (92.1-97.8)	53.5% (50.5-56.5)	84.2% (81.2-87.2)	56.2% (53.2-59.2)	84.6% (81.6-87.6)	29% (26-32)	73.8% (70.8-76.8)
**85-89**	49% (46-52)	77.6% (74.6-80.6)	66.3% (63.3-69.3)	94.9% (91.9-97.4)	54.7% (51.7-57.7)	83.6% (80.6-86.6)	57.4% (54.4-60.4)	83.9% (80.9-86.9)	30% (27-33)	72.9% (69.9-75.9)
**90-95**	50.2% (47.2-53.2)	76.8% (73.8-79.8)	67.4% (64.4-70.4)	94.6% (91.6-97.1)	55.9% (52.9-58.9)	82.9% (79.9-85.9)	58.6% (55.6-61.6)	83.2% (80.2-86.2)	31.1% (28.1-34.1)	71.9% (68.9-74.9)

Supplemental Table 38: Positive and Negative Predictive values of plasma biomarkers for amyloid-β pathology in individuals with Corticobasal syndrome using the lower estimate of amyloid-β pathology prevalence.

	p-tau181		p-tau217		p-tau231		GFAP		NfL	
**Age**	PPV (95%CI)	NPV (95%CI)	PPV (95%CI)	NPV (95%CI)	PPV (95%CI)	NPV (95%CI)	PPV (95%CI)	NPV (95%CI)	PPV (95%CI)	NPV (95%CI)
**50-54**	Prevalence data unavailable	Prevalence data unavailable	Prevalence data unavailable	Prevalence data unavailable	Prevalence data unavailable	Prevalence data unavailable	Prevalence data unavailable	Prevalence data unavailable	Prevalence data unavailable	Prevalence data unavailable
**55-59**	51.7% (48.7-54.7)	75.6% (72.6-78.6)	68.7% (65.7-71.7)	94.3% (91.3-97.3)	57.4% (54.4-60.4)	82% (79-85)	60.1% (57.1-63.1)	82.3% (79.3-85.3)	32.4% (29.4-35.4)	70.6% (67.6-73.6)
**60-64**	49.3% (46.3-52.3)	77.4% (74.4-80.4)	66.6% (63.6-69.6)	94.8% (91.8-97.8)	55% (52-58)	83.4% (80.4-86.4)	57.8% (54.8-60.8)	83.7% (80.7-86.7)	30.3% (27.3-33.3)	72.6% (69.6-75.6)
**65-69**	46.8% (43.8-49.8)	79.1% (76.1-82.1)	64.3% (61.3-67.3)	95.3% (92.3-98.3)	52.5% (49.5-55.5)	84.7% (81.7-87.7)	55.3% (52.3-58.3)	85% (82-88)	28.2% (25.2-31.2)	74.6% (71.6-77.6)
**70-74**	42.7% (39.7-45.7)	81.7% (78.7-84.7)	60.5% (57.5-63.5)	96% (93-99)	48.4% (45.4-51.4)	86.7% (83.7-89.7)	51.2% (48.2-54.2)	87% (84-90)	25% (22-28)	77.5% (74.5-80.5)
**75-79**	52.6% (49.6-55.6)	75% (72-78)	69.4% (66.4-72.4)	94.1% (91.1-97.1)	58.2% (55.2-61.2)	81.5% (78.5-84.5)	60.9% (57.9-63.9)	81.8% (78.8-84.8)	33.1% (30.1-36.1)	69.9% (66.9-72.9)
**80-84**	26.9% (23.9-29.9)	90% (87-93)	43% (40-46)	98% (95-101)	31.7% (28.7-34.7)	93% (90-96)	34.1% (31.1-37.1)	93.1% (90.1-96.1)	14.2% (11.2-17.2)	87.5% (84.5-90.5)
**85-89**	Prevalence data unavailable	Prevalence data unavailable	Prevalence data unavailable	Prevalence data unavailable	Prevalence data unavailable	Prevalence data unavailable	Prevalence data unavailable	Prevalence data unavailable	Prevalence data unavailable	Prevalence data unavailable
**90-95**	Prevalence data unavailable	Prevalence data unavailable	Prevalence data unavailable	Prevalence data unavailable	Prevalence data unavailable	Prevalence data unavailable	Prevalence data unavailable	Prevalence data unavailable	Prevalence data unavailable	Prevalence data unavailable

Supplemental Table 39: Positive and Negative Predictive values of plasma biomarkers for amyloid-β pathology in individuals with MCI using the upper estimate of amyloid-β pathology prevalence.

	**p-tau181**		**p-tau217**		**p-tau231**		**GFAP**		**NfL**	
**Age**	PPV (95%CI)	NPV (95%CI)	PPV (95%CI)	NPV (95%CI)	PPV (95%CI)	NPV (95%CI)	PPV (95%CI)	NPV (95%CI)	PPV (95%CI)	NPV (95%CI)
**50-54**	60.2% (57.2-)63.2	68.8% (65.8-71.8)	75.6% (72.6-78.6)	92.1% (89.1-95.1)	65.6% (62.6-68.6)	76.3% (73.3-79.3)	68% (65-71)	76.8% (73.8-79.8)	40.3% (37.3-43.3)	63% (60-66)
**55-59**	64.7% (61.7-)67.7	64.5% (61.5-67.5)	79% (76-82)	90.6% (87.6-93.6)	69.7% (66.7-72.7)	72.7% (69.7-75.7)	72% (69-75)	73.2% (70.2-76.2)	45% (42-48)	58.5% (55.5-61.5)
**60-64**	68.5% (65.5-)71.5	60.4% (57.4-63.4)	81.7% (78.7-84.7)	89% (86-92)	73.3% (70.3-76.3)	69.1% (66.1-72.1)	75.4% (72.4-78.4)	69.6% (66.6-72.6)	49.4% (46.4-52.4)	54.2% (51.2-57.2)
**65-69**	72.2% (69.2-)75.2	56.2% (53.2-59.2)	84.2% (81.2-87.2)	87.2% (84.2-90.2)	76.6% (73.6-79.6)	65.2% (62.2-68.2)	78.5% (75.5-81.5)	65.8% (62.8-68.8)	53.8% (50.8-56.8)	49.8% (46.8-52.8)
**70-74**	75.9% (72.9-)78.9	51.4% (48.4-54.4)	86.6% (83.6-89.6)	84.9% (81.9-87.9)	79.9% (76.9-82.9)	60.7% (57.7-63.7)	81.6% (78.6-84.6)	61.3% (58.3-64.3)	58.5% (55.5-61.5)	45% (42-48)
**75-79**	79.5% (76.5-)82.5	46.2% (43.2-49.2)	88.8% (85.8-91.8)	82% (79-85)	83% (80-86)	55.7% (52.7-58.7)	84.5% (81.5-87.5)	56.3% (53.3-59.3)	63.4% (60.4-66.4)	39.9% (36.9-42.9)
**80-84**	83% (80-)86	40.5% (37.5-43.5)	90.9% (87.9-93.9)	78.3% (75.3-81.3)	86% (83-89)	49.9% (46.9-52.9)	87.3% (84.3-90.3)	50.5% (47.5-53.5)	68.7% (65.7-71.7)	34.5% (31.5-37.5)
**85-89**	86.3% (83.3-)89.3	34.5% (31.5-37.5)	92.8% (89.8-95.8)	73.7% (70.7-76.7)	88.8% (85.8-91.8)	43.6% (40.6-46.6)	89.9% (86.9-92.9)	44.2% (41.2-47.2)	73.8% (70.8-76.8)	29% (26-32)
**90-95**	88.7% (85.7-)91.7	29.9% (26.9-32.9)	94.1% (91.1-97.1)	69.4% (66.4-72.4)	90.8% (87.8-93.8)	38.4% (35.4-41.4)	91.7% (88.7-94.7)	39% (36-42)	77.7% (74.7-80.7)	24.8% (21.8-27.8)

Supplemental Table 40: Positive and Negative Predictive values of plasma biomarkers for amyloid-β pathology in individuals with probable AD dementia using the upper estimate of amyloid-β pathology prevalence.

	p-tau181		p-tau217		p-tau231		GFAP		NfL	
**Age**	PPV (95%CI)	NPV (95%CI)	PPV (95%CI)	NPV (95%CI)	PPV (95%CI)	NPV (95%CI)	PPV (95%CI)	NPV (95%CI)	PPV (95%CI)	NPV (95%CI)
**50-54**	97.3% (94.3-98.9)	8.3% (5.3-11.3)	98.7% (95.7-99.2)	32.5% (29.5-35.5)	97.9% (94.9-98.2)	11.7% (8.7-14.7)	98.1% (95.1-98.5)	12% (9-15)	94.3% (91.3-97.3)	6.6% (3.6-9.6)
**55-59**	96.9% (93.9-99.9)	9.6% (6.6-12.6)	98.5% (95.5-99.0)	36.1% (33.1-39.1)	97.5% (94.5-98.1)	13.5% (10.5-16.5)	97.8% (94.8-98.1)	13.7% (10.7-16.7)	93.3% (90.3-96.3)	7.6% (4.6-10.6)
**60-64**	96.5% (93.5-99.5)	10.9% (7.9-13.9)	98.2% (95.2-98.7)	39.4% (36.4-42.4)	97.2% (94.2-97.9)	15.2% (12.2-18.2)	97.5% (94.5-97.9)	15.5% (12.5-18.5)	92.4% (89.4-95.4)	8.6% (5.6-11.6)
**65-69**	96% (93-99)	12.2% (9.2-15.2)	98% (95-98.6)	42.4% (39.4-45.4)	96.8% (93.8-99.5)	16.8% (13.8-19.8)	97.1% (94.1-97.4)	17.2% (14.2-20.2)	91.5% (88.5-94.5)	9.7% (6.7-12.7)
**70-74**	95.6% (92.6-98.6)	13.3% (10.3-16.3)	97.8% (94.8-98.3)	44.9% (41.9-47.9)	96.5% (93.5-99.3)	18.3% (15.3-21.3)	96.8% (93.8-97.2)	18.7% (15.7-21.7)	90.7% (87.7-93.7)	10.6% (7.6-13.6)
**75-79**	95.2% (92.2-98.2)	14.3% (11.3-17.3)	97.6% (94.6-98.1)	47% (44-50)	96.2% (93.2-99.0)	19.6% (16.6-22.6)	96.6% (93.6-97.0)	20% (17-23)	89.9% (86.9-92.9)	11.4% (8.4-14.4)
**80-84**	94.9% (91.9-97.9)	15% (12-18)	97.5% (94.5-98.0)	48.5% (45.5-51.5)	95.9% (92.9-98.6)	20.6% (17.6-23.6)	96.4% (93.4-96.8)	21% (18-24)	89.4% (86.4-92.4)	12.1% (9.1-15.1)
**85-89**	94.8% (91.8-97.8)	15.5% (12.5-18.5)	97.4% (94.4-98.0)	49.5% (46.5-52.5)	95.8% (92.8-98.5)	21.2% (18.2-24.2)	96.2% (93.2-96.6)	21.6% (18.6-24.6)	89% (86-92)	12.5% (9.5-15.5)
**90-95**	94.6% (91.6-97.6)	15.9% (12.9-18.9)	97.3% (94.3-97.9)	50.2% (47.2-53.2)	95.7% (92.7-98.3)	21.7% (18.7-24.7)	96.1% (93.1-96.5)	22.1% (19.1-25.1)	88.7% (85.7-91.7)	12.8% (9.8-15.8)

Supplemental Table 41: Positive and Negative Predictive values of plasma biomarkers for amyloid-β pathology in individuals with frontotemporal dementia using the lower estimate of amyloid-β pathology prevalence.

	p-tau181		p-tau217		p-tau231		GFAP		NfL	
**Age**	PPV (95%CI)	NPV (95%CI)	PPV (95%CI)	NPV (95%CI)	PPV (95%CI)	NPV (95%CI)	PPV (95%CI)	NPV (95%CI)	PPV (95%CI)	NPV (95%CI)
**50-54**	28.7% (25.7-31.7)	89.2% (86.2-92.2)	45.2% (42.2-48.2)	97.8% (94.8-98.1)	33.6% (30.6-36.6)	92.4% (89.4-95.4)	36.1% (33.1-39.1)	92.6% (89.6-95.6)	15.2% (12.2-18.2)	86.5% (83.5-89.5)
**55-59**	29.3% (26.3-32.3)	88.9% (85.9-91.9)	46% (43-49)	97.7% (94.7-98.0)	34.3% (31.3-37.3)	92.2% (89.2-95.2)	36.9% (33.9-39.9)	92.3% (89.3-95.3)	15.7% (12.7-18.7)	86.1% (83.1-89.1)
**60-64**	30.3% (27.3-33.3)	88.4% (85.4-91.4)	47.2% (44.2-50.2)	97.6% (94.6-97.9)	35.4% (32.4-38.4)	91.8% (88.8-94.8)	38% (35-41)	92% (89-95)	16.3% (13.3-19.3)	85.5% (82.5-88.5)
**65-69**	32% (29-35)	87.6% (84.6-90.6)	49.1% (46.1-52.1)	97.4% (94.4-97.7)	37.2% (34.2-40.2)	91.2% (88.2-94.2)	39.8% (36.8-42.8)	91.4% (88.4-94.4)	17.4% (14.4-20.4)	84.6% (81.6-87.6)
**70-74**	35.1% (32.1-38.1)	86% (83-89)	52.6% (49.6-55.6)	97% (94-97.5)	40.5% (37.5-43.5)	90% (87-93)	43.2% (40.2-46.2)	90.2% (87.2-93.2)	19.5% (16.5-22.5)	82.6% (79.6-85.6)
**75-79**	43.8% (40.8-46.8)	81% (78-84)	61.5% (58.5-64.5)	95.8% (92.8-97.3)	49.5% (46.5-52.5)	86.2% (83.2-89.2)	52.3% (49.3-55.3)	86.5% (83.5-89.5)	25.8% (22.8-28.8)	76.8% (73.8-79.8)
**80-84**	52.6% (49.6-55.6)	75% (72-78)	69.4% (66.4-72.4)	94.1% (91.1-97.1)	58.2% (55.2-61.2)	81.5% (78.5-84.5)	60.9% (57.9-63.9)	81.8% (78.8-84.8)	33.1% (30.1-36.1)	69.9% (66.9-72.9)
**85-89**	62.2% (59.2-65.2)	66.9% (63.9-69.9)	77.1% (74.1-80.1)	91.5% (88.5-94.5)	67.4% (64.4-70.4)	74.8% (71.8-77.8)	69.8% (66.8-72.8)	75.2% (72.2-78.2)	42.4% (39.4-45.4)	61% (58-64)
**90-95**	Prevalence data unavailable	Prevalence data unavailable	Prevalence data unavailable	Prevalence data unavailable	Prevalence data unavailable	Prevalence data unavailable	Prevalence data unavailable	Prevalence data unavailable	Prevalence data unavailable	Prevalence data unavailable

Supplemental Table 42: Positive and Negative Predictive values of plasma biomarkers for amyloid-β pathology in individuals with Vascular dementia using the upper estimate of amyloid-β pathology prevalence.

	p-tau181		p-tau217		p-tau231		GFAP		NfL	
**Age**	PPV (95%CI)	NPV (95%CI)	PPV (95%CI)	NPV (95%CI)	PPV (95%CI)	NPV (95%CI)	PPV (95%CI)	NPV (95%CI)	PPV (95%CI)	NPV (95%CI)
**50-54**	Prevalence data unavailable	Prevalence data unavailable	Prevalence data unavailable	Prevalence data unavailable	Prevalence data unavailable	Prevalence data unavailable	Prevalence data unavailable	Prevalence data unavailable	Prevalence data unavailable	Prevalence data unavailable
**55-59**	Prevalence data unavailable	Prevalence data unavailable	Prevalence data unavailable	Prevalence data unavailable	Prevalence data unavailable	Prevalence data unavailable	Prevalence data unavailable	Prevalence data unavailable	Prevalence data unavailable	Prevalence data unavailable
**60-64**	54.9% (51.9-57.9)	73.3% (70.3-76.3)	71.4% (68.4-74.4)	93.6% (90.6-96.6)	60.5% (57.5-63.5)	80% (77-83)	63.1% (60.1-66.1)	80.4% (77.4-83.4)	35.2% (32.2-38.2)	67.9% (64.9-70.9)
**65-69**	66% (63-69)	63.2% (60.2-66.2)	79.9% (76.9-82.9)	90.1% (87.1-93.1)	70.9% (67.9-73.9)	71.5% (68.5-74.5)	73.2% (70.2-76.2)	72% (69-75)	46.4% (43.4-49.4)	57.1% (54.1-60.1)
**70-74**	57% (54-60)	71.5% (68.5-74.5)	73.1% (70.1-76.1)	93% (90-96)	62.6% (59.6-65.6)	78.6% (75.6-81.6)	65.1% (62.1-68.1)	79% (76-82)	37.3% (34.3-40.3)	66% (63-69)
**75-79**	60.2% (57.2-63.2)	68.8% (65.8-71.8)	75.6% (72.6-78.6)	92.1% (89.1-95.1)	65.6% (62.6-68.6)	76.3% (73.3-79.3)	68% (65-71)	76.8% (73.8-79.8)	40.3% (37.3-43.3)	63% (60-66)
**80-84**	67.8% (64.8-70.8)	61.3% (58.3-64.3)	81.2% (78.2-84.2)	89.4% (86.4-92.4)	72.6% (69.6-75.6)	69.9% (66.9-72.9)	74.7% (71.7-77.7)	70.4% (67.4-73.4)	48.5% (45.5-51.5)	55.1% (52.1-58.1)
**85-89**	74.3% (71.3-77.3)	53.5% (50.5-56.5)	85.6% (82.6-88.6)	85.9% (82.9-88.9)	78.5% (75.5-81.5)	62.7% (59.7-65.7)	80.3% (77.3-83.3)	63.3% (60.3-66.3)	56.4% (53.4-59.4)	47.1% (44.1-50.1)
**90-95**	81.4% (78.4-84.4)	43.1% (40.1-46.1)	90% (87-93)	80.1% (77.1-83.1)	84.7% (81.7-87.7)	52.6% (49.6-55.6)	86.1% (83.1-89.1)	53.2% (50.2-56.2)	66.2% (63.2-69.2)	37% (34-40)

Supplemental Table 43: Positive and Negative Predictive values of plasma biomarkers for amyloid-β pathology in individuals with Corticobasal syndrome using the upper estimate of amyloid-β pathology prevalence.

	p-tau181		p-tau217		p-tau231		GFAP		NfL	
**Age**	PPV (95%CI)	NPV (95%CI)	PPV (95%CI)	NPV (95%CI)	PPV (95%CI)	NPV (95%CI)	PPV (95%CI)	NPV (95%CI)	PPV (95%CI)	NPV (95%CI)
**50-54**	Prevalence data unavailable	Prevalence data unavailable	Prevalence data unavailable	Prevalence data unavailable	Prevalence data unavailable	Prevalence data unavailable	Prevalence data unavailable	Prevalence data unavailable	Prevalence data unavailable	Prevalence data unavailable
**55-59**	82.1% (79.1-85.1)	42.1% (39.1-45.1)	90.4% (87.4-93.4)	79.4% (76.4-82.4)	85.2% (82.2-88.2)	51.5% (48.5-54.5)	86.6% (83.6-89.6)	52.2% (49.2-55.2)	67.2% (64.2-70.2)	36% (33-39)
**60-64**	78.7% (75.7-81.7)	47.4% (44.4-50.4)	88.4% (85.4-91.4)	82.7% (79.7-85.7)	82.3% (79.3-85.3)	56.8% (53.8-59.8)	83.9% (80.9-86.9)	57.4% (54.4-60.4)	62.3% (59.3-65.3)	41% (38-44)
**65-69**	75.1% (72.1-78.1)	52.5% (49.5-55.5)	86.1% (83.1-89.1)	85.4% (82.4-88.4)	79.1% (76.1-82.1)	61.8% (58.8-64.8)	80.9% (77.9-83.9)	62.4% (59.4-65.4)	57.4% (54.4-60.4)	46.1% (43.1-49.1)
**70-74**	70.3% (67.3-73.3)	58.4% (55.4-61.4)	82.9% (79.9-85.9)	88.2% (85.2-91.2)	74.9% (71.9-77.9)	67.3% (64.3-70.3)	76.9% (73.9-79.9)	67.8% (64.8-70.8)	51.5% (48.5-54.5)	52.1% (49.1-55.1)
**75-79**	71.2% (68.2-74.2)	57.4% (54.4-60.4)	83.5% (80.5-86.5)	87.8% (84.8-90.8)	75.6% (72.6-78.6)	66.4% (63.4-69.4)	77.6% (74.6-80.6)	66.9% (63.9-69.9)	52.5% (49.5-55.5)	51.1% (48.1-54.1)
**80-84**	72% (69-75)	56.5% (53.5-59.5)	84% (81-87)	87.3% (84.3-90.3)	76.4% (73.4-79.4)	65.5% (62.5-68.5)	78.3% (75.3-81.3)	66% (63-69)	53.5% (50.5-56.5)	50.1% (47.1-53.1)
**85-89**	Prevalence data unavailable	Prevalence data unavailable	Prevalence data unavailable	Prevalence data unavailable	Prevalence data unavailable	Prevalence data unavailable	Prevalence data unavailable	Prevalence data unavailable	Prevalence data unavailable	Prevalence data unavailable
**90-95**	Prevalence data unavailable	Prevalence data unavailable	Prevalence data unavailable	Prevalence data unavailable	Prevalence data unavailable	Prevalence data unavailable	Prevalence data unavailable	Prevalence data unavailable	Prevalence data unavailable	Prevalence data unavailable

Supplemental Table 44: Positive and Negative Predictive values of plasma biomarkers for amyloid-β pathology in individuals with Mild Cognitive Impairment using amyloid-β positivity prevalence estimates from a community-based setting.

	p-tau181		p-tau217		p-tau231		GFAP		NfL	
**Age**	PPV (95%CI)	NPV (95%CI)	PPV (95%CI)	NPV (95%CI)	PPV (95%CI)	NPV (95%CI)	PPV (95%CI)	NPV (95%CI)	PPV (95%CI)	NPV (95%CI)
**60-64**	39.6%	83.5%	57.3%	96.4%	45.2%	88.1%	48.0%	88.4%	22.7%	79.7%
**65-69**	48.9%	77.6%	66.3%	94.9%	54.7%	83.6%	57.4%	83.9%	30.0%	72.9%
**70-74**	62.1%	66.9%	77.1%	91.5%	67.4%	74.8%	69.8%	75.2%	42.4%	61.0%
**75-79**	71.1%	57.4%	83.5%	87.8%	75.6%	66.4%	77.6%	66.9%	52.5%	51.1%
**80-84**	81.4%	43.1%	90.0%	80.1%	84.7%	52.6%	86.1%	53.2%	66.2%	37.0%
**85-89**	87.5%	32.1%	93.5%	71.6%	89.8%	41.0%	90.8%	41.6%	75.8%	26.8%
**90-95**	91.8%	22.8%	95.8%	61.2%	93.4%	30.2%	94.1%	30.8%	83.4%	18.6%

Supplemental Table 45: Proportion of individuals with Mild Cognitive Impairment who are amyloid-β positive in each cohort.

Cohort (ref)	% of individuals with MCI who are amyloid-β positive
ADNI [4]	55.5%
BICWALZS [7]	27.0%
BioCogBank [41]	48.9%
BIODEGMAR [10]	59.7%
BioFINDER-2 [12]	60.3%
HABS-HD [18]	23.8%
MCSA [23]	55.4%
McGill Memory Clinic [26]	51.9%
SPIN [27]	45%
TRIAD [33]	59.2%

References

1. Petersen RC. Mild cognitive impairment as a diagnostic entity. In: Journal of Internal Medicine. 2004. p. 183–94.
2. McKhann G, Drachman D, Folstein M, Katzman R. Clinical diagnosis of Alzheimer's disease. Neurology 1984;34(7):939.
3. Shaw LM, Vanderstichele H, Knapik-Czajka M, et al. Cerebrospinal fluid biomarker signature in alzheimer's disease neuroimaging initiative subjects. Ann Neurol 2009;65(4):403–13.
4. Karikari TK, Benedet AL, Ashton NJ, et al. Diagnostic performance and prediction of clinical progression of plasma phospho-tau181 in the Alzheimer's Disease Neuroimaging Initiative. Mol Psychiatry 2020;181:429–42.
5. Morris JC. The Clinical Dementia Rating (CDR): current version and scoring rules. Neurology 1993;43(11):2412–4.
6. McKhann G, Knopman DS, Chertkow H, et al. The diagnosis of dementia due to Alzheimer's disease: Recommendations from the National Institute on Aging-Alzheimer's Association workgroups on diagnostic guidelines for Alzheimer's disease. Alzheimers Dement 2011;7(3):263–9.
7. Karim HT, Aizenstein HJ, Mizuno A, et al. Independent replication of advanced brain age in mild cognitive impairment and dementia: detection of future cognitive dysfunction. Mol Psychiatry 2022;27(12):5235–43.
8. Ashton NJ, Brum WS, Molfetta G Di, et al. Diagnostic accuracy of the plasma ALZpath pTau217 immunoassay to identify Alzheimer ' s disease pathology. MedXriv 2023;
9. Benedet AL, Milà-Alomà M, Vrillon A, et al. Differences between Plasma and Cerebrospinal Fluid Glial Fibrillary Acidic Protein Levels across the Alzheimer Disease Continuum. JAMA Neurol 2021;78(12):1471–83.
10. Ashton NJ, Puig-pijoan A, Milà-alomà M, et al. Plasma and CSF biomarkers in a memory clinic : Head-to-head comparison of phosphorylated tau immunoassays. Alzheimer's Dement 2022;(July):1–12.
11. Palmqvist S, Rossi M, Hall S, et al. Cognitive effects of Lewy body pathology in clinically unimpaired individuals. Nat Med 2023;
12. Palmqvist S, Janelidze S, Quiroz YT, et al. Discriminative Accuracy of Plasma Phospho-tau217 for Alzheimer Disease vs Other Neurodegenerative Disorders. JAMA 2020;324(8):772–81.
13. Janelidze S, Bali D, Ashton NJ, et al. Head-to-head comparison of 10 plasma phospho-tau assays in prodromal Alzheimer's disease. Brain 2022;1–6.
14. Quadalti C, Palmqvist S, Hall S, et al. Clinical effects of Lewy body pathology in cognitively impaired individuals. Nat Med 2023;
15. Hansson O, Batrla R, Brix B, et al. The Alzheimer's Association international guidelines for handling of cerebrospinal fluid for routine clinical measurements of amyloid β and tau. Alzheimer's Dement 2021;17(9):1575–82.
16. Gobom J, Parnetti L, Rosa-Neto P, et al. Validation of the LUMIPULSE automated immunoassay for the measurement of core AD biomarkers in cerebrospinal fluid. Clin Chem Lab Med 2022;60(2):207–19.
17. Leuzy A, Smith R, Cullen NC, et al. Biomarker-Based Prediction of Longitudinal Tau Positron Emission Tomography in Alzheimer Disease. JAMA Neurol 2022;79(2):149–58.

18. O'bryant SE, Johnson LA, Barber RC, et al. The health & aging brain among latino elders (Hable) study methods and participant characteristics. Alzheimer's Dement Diagnosis, Assess Dis Monit 2021;13(1):1–10.
19. Hall JR, Petersen M, Johnson L, O'Bryant SE. Characterizing Plasma Biomarkers of Alzheimer's in a Diverse Community-Based Cohort: A Cross-Sectional Study of the HAB-HD Cohort. Front Neurol 2022;13(August):1–8.
20. Jack CR, Therneau TM, Weigand SD, et al. Prevalence of Biologically vs Clinically Defined Alzheimer Spectrum Entities Using the National Institute on Aging-Alzheimer's Association Research Framework. JAMA Neurol 2019;76(10):1174–83.
21. Jack CR, Wiste HJ, Weigand SD, et al. Defining imaging biomarker cut points for brain aging and Alzheimer's disease. Alzheimer's Dement [Internet] 2017;13(3):205–16. Available from: http://dx.doi.org/10.1016/j.jalz.2016.08.005
22. Lowe VJ, Lundt ES, Albertson SM, et al. Tau-positron emission tomography correlates with neuropathology findings. Alzheimer's Dement 2020;16(3):561–71.
23. Mielke MM, Dage JL, Frank RD, et al. Performance of plasma phosphorylated tau 181 and 217 in the community. Nat Med 2022;
24. Shaw LM, Arias J, Blennow K, et al. Appropriate use criteria for lumbar puncture and cerebrospinal fluid testing in the diagnosis of Alzheimer's disease. Alzheimer's Dement [Internet] 2018;14(11):1505–21. Available from: https://doi.org/10.1016/j.jalz.2018.07.220
25. Johnson KA, Minoshima S, Bohnen NI, et al. Appropriate use criteria for amyloid PET: a report of the Amyloid Imaging Task Force, the Society of Nuclear Medicine and Molecular Imaging, and the Alzheimer's Association. Alzheimers Dement 2013;54(3):476–90.
26. Quispialaya KM, Therriault J, Aliaga A, et al. Discordance and Concordance between Cerebrospinal and [18F]FDG-PET Biomarkers in Assessing Atypical and Early-Onset AD Dementia Cases. Neurology 2022;99(22):E2428–36.
27. Alcolea D, Clarimón J, Carmona-Iragui M, et al. The Sant Pau Initiative on Neurodegeneration (SPIN) cohort: A data set for biomarker discovery and validation in neurodegenerative disorders. Alzheimer's Dement Transl Res Clin Interv 2019;5:597–609.
28. Mattsson N, Andreasson U, Persson S, et al. The Alzheimer's Association external quality control program for cerebrospinal fluid biomarkers. Alzheimer's Dement 2011;7(4):386–95.
29. Alcolea D, Pegueroles J, Muñoz L, et al. Agreement of amyloid PET and CSF biomarkers for Alzheimer's disease on Lumipulse. Ann Clin Transl Neurol 2019;6(9):1815–24.
30. Lleó A, Zetterberg H, Pegueroles J, et al. Phosphorylated tau181 in plasma as a potential biomarker for Alzheimer's disease in adults with Down syndrome. Nat Commun [Internet] 2021;12(1):1–8. Available from: http://dx.doi.org/10.1038/s41467-021-24319-x
31. Montoliu-Gaya L, Alcolea D, Ashton NJ, et al. Plasma and cerebrospinal fluid glial fibrillary acidic protein levels in adults with Down syndrome: a longitudinal cohort study. eBioMedicine [Internet] 2023;90:104547. Available from: https://doi.org/10.1016/j.ebiom.2023.104547
32. Carmona-Iragui M, Alcolea D, Barroeta I, et al. Diagnostic and prognostic performance and longitudinal changes in plasma neurofilament light chain concentrations in adults with Down syndrome: a cohort study. Lancet Neurol [Internet] 2021;20(8):605–14. Available

from: http://dx.doi.org/10.1016/S1474-4422(21)00129-0
33. Therriault J, Benedet AL, Pascoal TA, et al. Association of Apolipoprotein e ϵ4 with Medial Temporal Tau Independent of Amyloid-β. JAMA Neurol 2020;77(4):470–9.
34. Therriault J, Pascoal TA, Lussier FZ, et al. Biomarker modeling of Alzheimer's disease using PET-based Braak staging. Nat Aging 2022;
35. Therriault J, Servaes S, Tissot C, et al. Equivalence of plasma p-tau217 with cerebrospinal fluid in the diagnosis of Alzheimer's disease. Alzheimer's Dement 2023;(January):1–11.
36. Smirnov DS, Ashton NJ, Blennow K, et al. Plasma biomarkers for Alzheimer's Disease in relation to neuropathology and cognitive change. Acta Neuropathol [Internet] 2022;143(4):487–503. Available from: https://doi.org/10.1007/s00401-022-02408-5
37. Mirra SS, Heyman A, McKeel D, et al. The Consortium to Establish a Registry for Alzheimer's Disease (CERAD). - Part II. Standardization of the neuropathologic assessment of Alzheimer's disease. Neurology 1991;41(April):479–86.
38. Braak H, Alafuzoff I, Arzberger T, Kretzschmar H, Tredici K. Staging of Alzheimer disease-associated neurofibrillary pathology using paraffin sections and immunocytochemistry. Acta Neuropathol 2006;112(4):389–404.
39. Ashton NJ, Pascoal TA, Karikari TK, et al. Plasma p-tau231: a new biomarker for incipient Alzheimer's disease pathology. Acta Neuropathol [Internet] 2021;141(5):709–24. Available from: https://doi.org/10.1007/s00401-021-02275-6
40. Thijssen EH, La Joie R, Strom A, et al. Plasma phosphorylated tau 217 and phosphorylated tau 181 as biomarkers in Alzheimer's disease and frontotemporal lobar degeneration: a retrospective diagnostic performance study. Lancet Neurol [Internet] 2021;20(9):739–52. Available from: http://dx.doi.org/10.1016/S1474-4422(21)00214-3
41. Benedet AL, Milà-Alomà M, Vrillon A, et al. Differences between Plasma and Cerebrospinal Fluid Glial Fibrillary Acidic Protein Levels across the Alzheimer Disease Continuum. JAMA Neurol 2021;78(12):1471–83.
