# Supplementary material for: Diagnosis of Alzheimer’s disease using plasma biomarkers adjusted to clinical probability
Source: Nat Aging. 2024 Nov 12;4(11):1529–37. doi: 10.1038/s43587-024-00731-y (PMC11564087; doi:10.1038/s43587-024-00731-y)
Supplement: Supplementary file 2 — Reporting Summary [file 43587_2024_731_MOESM2_ESM.pdf]

nature portfolio

Corresponding author(s): Joseph Therriault

Last updated by author(s): Aug 7, 2024

Reporting Summary

Nature Portfolio wishes to improve the reproducibility of the work that we publish. This form provides structure for consistency and transparency in reporting. For further information on Nature Portfolio policies, see our Editorial Policies and the Editorial Policy Checklist.

Statistics

For all statistical analyses, confirm that the following items are present in the figure legend, table legend, main text, or Methods section.

n/a	Confirmed	
☐	☒	The exact sample size (n) for each experimental group/condition, given as a discrete number and unit of measurement
☐	☒	A statement on whether measurements were taken from distinct samples or whether the same sample was measured repeatedly
☐	☒	The statistical test(s) used AND whether they are one- or two-sided<br>*Only common tests should be described solely by name; describe more complex techniques in the Methods section.*
☐	☒	A description of all covariates tested
☒	☐	A description of any assumptions or corrections, such as tests of normality and adjustment for multiple comparisons
☐	☒	A full description of the statistical parameters including central tendency (e.g. means) or other basic estimates (e.g. regression coefficient) AND variation (e.g. standard deviation) or associated estimates of uncertainty (e.g. confidence intervals)
☒	☐	For null hypothesis testing, the test statistic (e.g. F, t, r) with confidence intervals, effect sizes, degrees of freedom and P value noted<br>*Give P values as exact values whenever suitable.*
☐	☒	For Bayesian analysis, information on the choice of priors and Markov chain Monte Carlo settings
☒	☐	For hierarchical and complex designs, identification of the appropriate level for tests and full reporting of outcomes
☒	☐	Estimates of effect sizes (e.g. Cohen's d, Pearson's r), indicating how they were calculated

Our web collection on statistics for biologists contains articles on many of the points above.

Software and code

Policy information about availability of computer code

Data collection	No specific code was used in data collection.
Data analysis	No custom code was used in the analysis of this study. The formulas used for the generation of the study's main results are provided in the methods section. Data were visualized using Graphpad Prism (version 10).

For manuscripts utilizing custom algorithms or software that are central to the research but not yet described in published literature, software must be made available to editors and reviewers. We strongly encourage code deposition in a community repository (e.g. GitHub). See the Nature Portfolio guidelines for submitting code & software for further information.

Data

Policy information about availability of data

All manuscripts must include a data availability statement. This statement should provide the following information, where applicable:

- Accession codes, unique identifiers, or web links for publicly available datasets
- A description of any restrictions on data availability
- For clinical datasets or third party data, please ensure that the statement adheres to our policy

Data availability statement
Data from the ADNI cohort can be accessed from https://ida.loni.usc.edu. Data from the HABS-HB study can be accessed from https://apps.unthsc.edu/itr/researchers. Raw and analyzed de-identified data from the Mayo Clinic Study of Aging can be requested at https://ras-rdrs.mayo.edu/Request/IndexRequest. The

request will be reviewed by the Mayo Clinic Study of Aging investigators and Mayo Clinic to verify whether the request is subject to any intellectual property or confidentiality obligations. A data sharing agreement must be obtained prior to release. Anonymized data from the BICWALZS, BioCogBank, BIODEGMAR, BioFINDER, SPIN, and UCSD-ADRC cohort studies will be shared by request from a qualified academic investigator for the sole purpose of replicating procedures and results presented in this Article and as long as the data transfer is in agreement with all local legislation on general data protection regulation and will be regulated by a material transfer agreement. Arrangements for data sharing for replication of the findings in the TRIAD data set are subject to standard data-sharing agreements, and further information can be found on the study's website (https://triad. tnl-mcgill.com/).

Research involving human participants, their data, or biological material

Policy information about studies with human participants or human data. See also policy information about sex, gender (identity/presentation), and sexual orientation and race, ethnicity and racism.

Reporting on sex and gender	We used the term "sex" in the manuscript, and sex was determined based on participant self-reporting. All cohorts included recruit both self-reported men and women. No sex-specific analyses were conducted.
Reporting on race, ethnicity, or other socially relevant groupings	A summary of the representativeness of the study participants is provided in Supplementary Table 1. Race and ethnicity were determined based on participant self-reporting. Furthermore, between-cohort differences in race and ethnicity are described in the discussion.
Population characteristics	Population characteristics are provided in Table 1. A cross-cohort comparison of key demographic features is provided in Supplementary Table 2, and population characteristics of each individual cohort study is provided in Supplementary Tables 3-14. Race and ethnicity data for all study participants together is reported in Supplementary Table 15.
Recruitment	Subjects were included from prospective cohort studies in Canada, France, South Korea, Spain, Sweden, and the United States. Subject recruitment protocols for each cohort study are provided in the supplementary material, on pages 2-13. Potential study biases related to related to recruitment are described in the limitations section of the discussion.
Ethics oversight	All study participants provided written informed consent and local institutional review boards approved the studies. Information on ethics approvals for each cohort is provided in the supplementary material on pages 2-13.

Note that full information on the approval of the study protocol must also be provided in the manuscript.

Field-specific reporting

Please select the one below that is the best fit for your research. If you are not sure, read the appropriate sections before making your selection.

☒ Life sciences ☐ Behavioural & social sciences ☐ Ecological, evolutionary & environmental sciences

For a reference copy of the document with all sections, see nature.com/documents/nr-reporting-summary-flat.pdf

Life sciences study design

All studies must disclose on these points even when the disclosure is negative.

Sample size	We included a total of 6896 individuals assessed with clinical assessments, plasma biomarkers and PET, CSF or neuropathological assessments. Sample size was not determined a priori and was instead determined based on availability of data. The prevalence data is based on meta-analyses of over 19,000 individuals, and therefore we believe it is unlikely that these data are underpowered.
Data exclusions	Young individuals (age < 25 years) were not included in TRIAD cohort analyses.
Replication	Results of this study were consistent across 11 observational cohort studies from 6 countries.
Randomization	This is an obervational diagnostic study and no allocation into groups was performed. Hence randomization is not relevant to this study.
Blinding	All fluid biomarker analyses were performed by individuals who were blinded to the clinical and CSF/PET data. Authors who performed the data pre-processing were blinded to demographic and clinical characteristics of study participants.

Reporting for specific materials, systems and methods

We require information from authors about some types of materials, experimental systems and methods used in many studies. Here, indicate whether each material, system or method listed is relevant to your study. If you are not sure if a list item applies to your research, read the appropriate section before selecting a response.

Materials & experimental systems

n/a	Involved in the study
☐	☒ Antibodies
☒	☐ Eukaryotic cell lines
☒	☐ Palaeontology and archaeology
☒	☐ Animals and other organisms
☒	☐ Clinical data
☒	☐ Dual use research of concern
☒	☐ Plants

Methods

n/a	Involved in the study
☒	☐ ChIP-seq
☒	☐ Flow cytometry
☒	☐ MRI-based neuroimaging

Antibodies

Antibodies used

Plasma phosphorylated tau 217 (p-tau217) and p-tau181 was quantified in BioFINDER-2, the Mayo Clinic Study of Aging, and the BIODEGMAR cohorts with immunoassays developed by Lilly Research Laboratories, and analyses were performed with the same batch of reagents. For p-tau217, biotinylated-IBA493 was used as a capture antibody and SULFO-TAG-4G102 as the detector. For p-tau181, AT270 was used as a capture antibody and SULFOTAG-4G102 as the detector. In TRIAD, plasma p-tau217 was quantified with an immunoassay developed by Janssen R&D, using the PT3 antibody as capture and HT43 as detector. In TRIAD, p-tau231 was measured with an immunoassay developed in-house by the University of Gothenburg, using Tau12 as detector and AT270 as capture. In all cohorts, p-tau231 was measured with an immunoassay developed in-house by the University of Gothenburg, using Tau12 as detector and ADx253 as capture. The HABS-HD study used the p-tau181 immunoassays developed by Lilly Research Laboratories.

Validation

The plasma p-tau217 immunoassay from Lilly has been previously validated and described in detail by Palmqvist et al. (JAMA. 2020:324(8):772-781) and by Groot et al (Alz Res Ther; 2022 May 14;14(1):67). The assay version of p-tau181 used in BioFINDER-2 has been previously validated by Janelidze et al (Nat Med; 2020 Mar;26(3):379-386). The plasma p-tau231 assay used in both cohorts has been previously validated by Ashton et al (Acta Neuropathol; 2021 May;141(5):709-724). In the Mayo Clinic Study of Aging, p-tau181 and p-tau217 were measured in duplicate on the MSD platform by electrochemiluminescence using proprietary assays from Lilly Research Laboratories, as described in Mielke et al (JAMA Neurology 2021). The ALZpath p-tau217 assay was validated as described in Ashton et al (JAMA Neurology 2023). Validation for the plasma assays in the BIODEGMAR study are described in Ashton et al (Alzheimer's and Dementia 2022). Validation of the plasma p-tau181 assay in the ADNI cohort, TRIAD cohort and BioCogBank cohorts is described in Karikari et al (Lancet Neurology 2020).The p-tau217 assay used in the TRIAD cohort has been validated in Triana-Baltzer et al (Alzheimer's & Dementia: Diagnosis, Assessment and disease monitoring 2021).

Plants

Seed stocks

Report on the source of all seed stocks or other plant material used. If applicable, state the seed stock centre and catalogue number. If plant specimens were collected from the field, describe the collection location, date and sampling procedures.

Novel plant genotypes

Describe the methods by which all novel plant genotypes were produced. This includes those generated by transgenic approaches, gene editing, chemical/radiation-based mutagenesis and hybridization. For transgenic lines, describe the transformation method, the number of independent lines analyzed and the generation upon which experiments were performed. For gene-edited lines, describe the editor used, the endogenous sequence targeted for editing, the targeting guide RNA sequence (if applicable) and how the editor was applied.

Authentication

Describe any authentication procedures for each seed stock used or novel genotype generated. Describe any experiments used to assess the effect of a mutation and, where applicable, how potential secondary effects (e.g. second site T-DNA insertions, mosiacism, off-target gene editing) were examined.
